# Ion–Conducting Ceramic Membrane Reactors for the Conversion of Chemicals

**DOI:** 10.3390/membranes13070621

**Published:** 2023-06-25

**Authors:** Zhicheng Zhang, Wanglin Zhou, Tianlei Wang, Zhenbin Gu, Yongfan Zhu, Zhengkun Liu, Zhentao Wu, Guangru Zhang, Wanqin Jin

**Affiliations:** 1 State Key Laboratory of Materials–Oriented Chemical Engineering, College of Chemical Engineering, Nanjing Tech University, 30 Puzhu Road(S), Nanjing 211816, China; zzc@njtech.edu.cn (Z.Z.); wlzhou@njtech.edu.cn (W.Z.); 201862104013@njtech.edu.cn (T.W.); guzhenbin@njtech.edu.cn (Z.G.); yongfanzhu@njtech.edu.cn (Y.Z.); liuzhengkun@njtech.edu.cn (Z.L.); wqjin@njtech.edu.cn (W.J.); 2 Energy and Bioproducts Research Institute (EBRI), Aston University, Birmingham B4 7ET, UK

**Keywords:** mixed conducting membranes, inorganic membranes, catalytic membrane reactors, heterogeneous reaction

## Abstract

Ion–conducting ceramic membranes, such as mixed oxygen ionic and electronic conducting (MIEC) membranes and mixed proton–electron conducting (MPEC) membranes, have the potential for absolute selectivity for specific gases at high temperatures. By utilizing these membranes in membrane reactors, it is possible to combine reaction and separation processes into one unit, leading to a reduction in by–product formation and enabling the use of thermal effects to achieve efficient and sustainable chemical production. As a result, membrane reactors show great promise in the production of various chemicals and fuels. This paper provides an overview of recent developments in dense ceramic catalytic membrane reactors and their potential for chemical production. This review covers different types of membrane reactors and their principles, advantages, disadvantages, and key issues. The paper also discusses the configuration and design of catalytic membrane reactors. Finally, the paper offers insights into the challenges of scaling up membrane reactors from experimental stages to practical applications.

## 1. Introduction

The chemical and petrochemical industries are vital to the global economy, but traditional reaction processes often require significant energy and additional facilities for the separation of mixtures of products, by–products, or raw materials. Advanced membrane reactor technology provides a solution by integrating separation and reaction processes. This means that the energy required to maintain the reaction, or the energy generated by the reaction can be harnessed to separate pure substances, including intermediate or final products. Membrane reactor technology offers a more efficient and sustainable approach to chemical and petrochemical production, making it an attractive option for reducing energy consumption and minimizing environmental impact.

The concept of catalytic membrane reactors (CMRs) was first introduced in the 1960s [1], and since then, significant progress has been made, particularly in the past two decades, as advancements in membrane processes have been made [2,3,4,5,6]. This progress has been facilitated by the development of new membrane materials, enabling membrane technology to be applied to a much broader range of operating conditions [7,8,9,10]. New configurations of membrane reactors and innovative reaction routes have also contributed to the development of membrane reactors. Inorganic membranes, including metals, ceramics, zeolites, glasses, and carbon, are commonly used in CMRs [11,12,13,14]. These membranes typically consist of multiple layers of single–phase or composite materials. Inorganic membranes offer a significant advantage over organic membranes because of their ability to operate at high temperatures and can be applied to more reactions. Inorganic membranes that possess high–temperature structural and chemical stability are potential candidates for CMRs.

Among the inorganic membranes, dense mixed conducting membranes are particularly noteworthy. These membranes include mixed oxygen ionic and electronic conducting (MIEC) and mixed proton–electron conducting (MPEC) membranes, which operate through a unique separation mechanism in which oxygen or hydrogen diffuses through the membrane in a dissociated or ionized form, rather than through conventional molecular diffusion or molecular sieve [15]. The most attractive feature of MIEC and MPEC membranes is their exceptional selectivity to oxygen and hydrogen, which makes them highly promising for applications in high–purity gas separation and CMRs.

MIEC membranes operate by dissociating oxygen molecules into oxygen ions, which are transported from the permeate side to the sweep side by receiving electrons. Oxygen ions then release electrons and regenerate oxygen molecules. Since the lattice can only contain and transfer oxygen ions, MIEC membranes can achieve high selectivity (up to 100%) without an external circuit [4,16,17]. On the other hand, MPEC membranes operate differently. Hydrogen molecules diffuse to and adsorb on the membrane surface, dissociating into hydrogen atoms. These atoms, easily losing their electrons, become protonic defects in the oxide lattice. Driven by the chemical potential gradient, the defects diffuse in the lattice from the feed side to the permeate side of the membrane and then recombine with electrons to form hydrogen molecules. The selectivity of MPEC membranes can theoretically reach 100%, but the presence of water vapor in the feed gas can lead to oxygen co–transportation, resulting in the permeated gas containing water vapor [18,19]. Ceramic MPEC membranes offer the advantage of being less expensive than Pd–based membranes, with relatively high durability and stability in CO–, CO_2_–, and H_2_S–containing water vapor environments [20].

**Figure 1:**
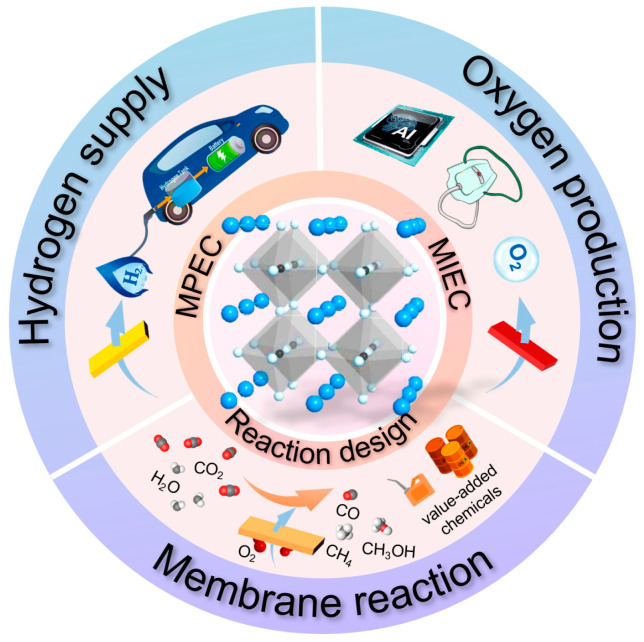
The main application of dense mixed conducting membranes.

In the past two decades, research on mixed conducting membranes has been driven by efforts to promote efficient energy utilization and reduce emissions, leading to the production of potential products such as oxygen, hydrogen, syngas, methane, ammonia, and higher hydrocarbons (mainly C2) [21,22,23,24,25,26] (Figure 1). Several major initiatives have made significant advances in bringing the technology closer to commercial readiness. Currently, research in this field is focused on identifying materials that combine high permeability with reliable chemical and thermal stability, optimizing step economy and atom economy reactions for membrane reactors, and developing new reaction routes to directly produce advanced chemicals, such as olefins and aromatics, in a membrane reactor. We realized that attempting a comprehensive review of this rapidly expanding and diverse field exceeds the scope of this article. Our objective is not to provide an exhaustive overview of all applications, and therefore, materials, theories, and modeling research will not be extensively discussed. Furthermore, a review of the many new developments in the field of solid electrolyte electrocatalytic reactors based on ion–conducting ceramic membranes is beyond the scope of this contribution. CMRs have been extensively reviewed in the past few years, including notable examples such as the review by Wang and Caro in 2013 [27], Sunarso and Liu in 2018 [28], and Yang in 2019 [29]. While previous reviews have typically focused on either MIEC or MPEC membranes, it is important to discuss these two membranes together. By considering their common features, such as the functions of the membrane, architectures, fabrication and scaling up strategies, readers can obtain a more comprehensive understanding of their potential applications and the design of new membrane reactors. Exploring these aspects collectively will provide valuable insights and foster innovation in the field.

## 2. Fundamentals

**Figure 2:**
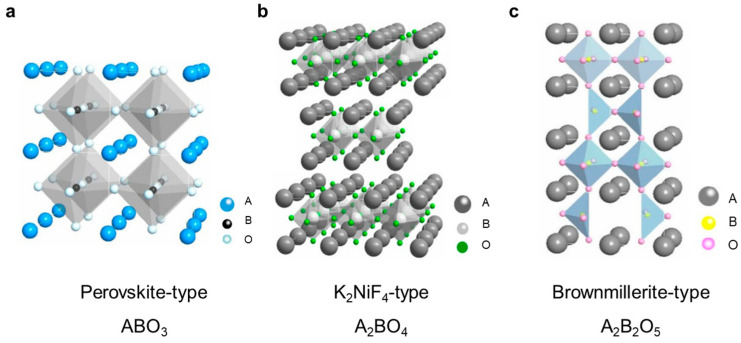
Crystal structures of three typical mixed conducting ceramic membrane materials: (**a**) Perovskite–type, (**b**) K_2_NiF_4_–type, (**c**) Brownmillerite–type. Reprinted with permission from Ref. [30]. Copyright 2013 China Science Press.

The performance of mixed conducting ceramic membranes is highly dependent on the properties of their constituent materials, which are mainly determined by their composition and structure. Among the various mixed conducting materials, perovskite oxides have been extensively studied due to their high–temperature stability and unique cubic or orthorhombic structure. Figure 2a illustrates the coordination environment of the A–site and B–site cations in a simple cubic perovskite structure, where the A–site cation coordinates with 12 oxygen ions to form a cubic octahedral coordination environment, while the B–site cation coordinates with six oxygen ions in an octahedral geometry. Typically, the A–site cation is larger than the B–site cation in perovskite, which helps to maintain the lattice stability. The perovskite structure provides a framework for preparing mixed conducting materials with desired chemical composition and structural characteristics. In addition to perovskite–type oxides, K_2_NiF_4_–type oxides (Figure 2b) and Brownmillerite–type oxides (Figure 2c) are also commonly employed for oxygen and hydrogen separations, as well as membrane reactions. Perovskite oxides are extensively studied as membrane materials, and therefore the review primarily delves into the exploration and analysis of perovskite oxides as a prominent category of materials for dense ceramic membranes.

The ideal perovskite structure is composed of ABO_3_ units, but its chemical composition can vary depending on the valence state of the A– and B–site cations. Common combinations include A^1+^B^5+^O_3_, A^2+^B^4+^O_3_, and A^3+^B^3+^O_3_. The A–site is typically occupied by large alkali earth metals like Ba, La, or Sr with a valence of 2+, and the B–site is occupied by first–row transition elements like Ce, Co, or Fe, with a valence of 4+. Despite their shared structure, perovskites with different compositions can exhibit distinct properties. For instance, BaCoO_3_ and SrCoO_3_ conduct oxygen, whereas BaCeO_3_ and SrCeO_3_ conduct protons. A stable perovskite structure relies on the existence of a stable BO_3_ skeletal sub–lattice [31], with the radius of the oxygen ion at 0.140 nm. To maintain a stable octahedral perovskite structure, the ionic radius of the B–site cation must be greater than 0.051 nm. A large A–site cation can further stabilize the BO_3_ skeletal sub–lattice by occupying the center of the eight BO_6_ octahedra. Although the presence of the A–site cation distorts the BO_3_ skeletal sub–lattice, it attempts to achieve optimal A metal cation–oxygen ion bond length, with a minimum radius of 0.09 nm for the A cation. Increased distortion can cause cubic crystal geometries to become orthorhombic or rhombohedral.

The tolerance factor *t* is used to analyze the relationship between ionic radius and crystal system, and is defined as $\frac{\left(R_{A}+R_{B}\right)}{\sqrt{{2R}_{O}}}$, where R_A_, R_B_, and R_O_ represent the ionic radii of the A–site cation (12–coordination), B–site cation (6–coordination), and oxygen ion, respectively. Cubic structures might exist between the limits of 0.75 < *t* < 1.0. For 0.75 < *t* < 0.9, the corner shared octahedra buckle cooperatively, leading to orthorhombic distortion. Orthorhombic distortion occurs when the BO_6_ octahedra are tilted, causing the A ions to be displaced along the (110) pseudo–cubic or (010) directions. For 0.75 < *t* < 0.9, a small deformation from cubic to rhombohedral symmetry may occur, without any octahedral buckling. The difference in the ionic radii of dopants and the outer electronic structure can easily cause distortion from the ideal cubic structure in perovskite oxides [32].

Mixed conducting materials are highly valued due to their ability to conduct both ions (or protons) and electrons, which enables the transport of oxygen or hydrogen through the material. To achieve optimal performance, it is important for the conductivity of both ions and electrons to be well matched. In many perovskite materials, electron conductivity is much greater than ion conductivity, whereas in many fluorite materials, the opposite is true. When electron conductivity is insufficient, an external circuit may be necessary to assist electronic conduction for effective ion transmission.

Ion doping is a common strategy for adjusting the physicochemical properties and improving both ion and electron conductivity. By introducing a lower valence dopant B’ on the B site of perovskite–structured oxides ABO_3_, AB_1−x_B’_x_O_3−δ_ can be formed, creating oxygen vacancies at high temperatures (represented by the symbol *δ*) that allow for ion transport [33]. Additionally, a wide range of perovskite–structured compounds can be produced by substituting A or B ions, or both, to form a structure of A_x_A’_1−x_B_y_B’_1−y_O_3−δ_.

The mobility of oxygen ions in the bulk phase is strongly influenced by A–site cations [34], while the nature of B–site cations determines the catalytic activity for reactions. Materials containing both types of cations can be used as membrane reactors that simultaneously serve as membranes for separating oxygen and catalysts for oxidation processes, eliminating the need for additional catalysts [35]. However, materials with high oxygen permeability but low catalytic activity can also be used for building membrane reactors, but additional catalysts are required [36,37,38,39].

## 3. Classification of Mixed Conducting Membranes

**Figure 3:**
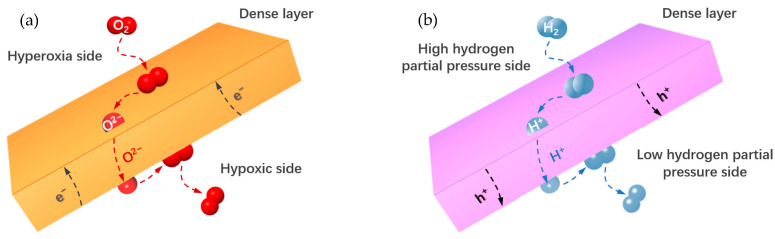
The permeation mechanism of (**a**) MIEC and (**b**) MPEC membranes.

MIEC and MPEC materials are categories of mixed conducting materials that can conduct both oxygen ions and electrons (Figure 3a), and protons and electrons (Figure 3b), respectively. These materials can be used to create membranes for oxygen or hydrogen separation and production, fuel cells, and gas or hydrogen sensors, among other applications. In the following sections, we will discuss typical materials used for oxygen–permeable and hydrogen–permeable membranes in greater detail.

### 3.1. Single–Phase Oxygen–Permeable Membranes

Fluorite–type oxides are the most commonly used materials for oxygen ionic conductors, with Yttria–stabilized zirconia (YSZ) being a well–known example that has been used for solid oxide fuel cells (SOFCs), sensors, and structural ceramics. However, the electron conductivity of YSZ is insufficient for oxygen permeation without an external circuit, leading to increased complexity and instability of the membrane reactor [40]. Therefore, fluorite–type oxides are typically used as oxygen ion–conducting materials in dual–phase membranes rather than as membranes alone.

K_2_NiF_4_–type oxides, on the other hand, are a type of mixed conducting material that has been studied for their potential use in oxygen–permeable membranes [41,42]. These materials have a unique structure that is composed of layers of edge–sharing MO_6_ octahedra and layers of corner–sharing MO_6_ octahedra, where M is a metal cation. The oxygen ions can move through the channels between these layers, allowing for high oxygen ionic conductivity. These K_2_NiF_4_–type composites do not contain alkaline–earth metal elements, which tend to react with CO_2_ to produce carbonates, and therefore have excellent stability against CO_2_ in comparison with perovskite oxides such as Pr_2_NiO_4+δ_ (PNO) and Pr_2_Ni_0.9_Mo_0.1_O_4+δ_ (PNM). With the doping of Mo, the oxygen permeability of the PNM hollow–fiber membrane is higher [43].

Perovskite oxides have been reported as highly ionic and electronic conducting materials for oxygen–permeable membranes, as firstly reported by Teraoka et al. [44]. These oxides possess a unique crystal structure that provides desirable transport properties, such as high ionic and electron conductivity. Furthermore, they can be easily synthesized with various compositions, allowing for tailoring of their properties to meet specific requirements for different applications. Additionally, perovskite oxides exhibit good chemical and thermal stability under harsh, high–temperature conditions, which is crucial for their long–term operation in various applications [33,45,46,47,48,49]. Researchers are continuing to develop new perovskite oxide compositions with enhanced properties, and to optimize synthesis and processing methods to improve the stability and reliability of the resulting membranes. Additionally, they are exploring new membrane architectures and configurations to further improve the performance and capabilities of perovskite oxide membranes for a range of applications. These will be discussed in detail in the following sections.

### 3.2. Dual–Phase Oxygen–Permeable membranes

**Figure 4:**
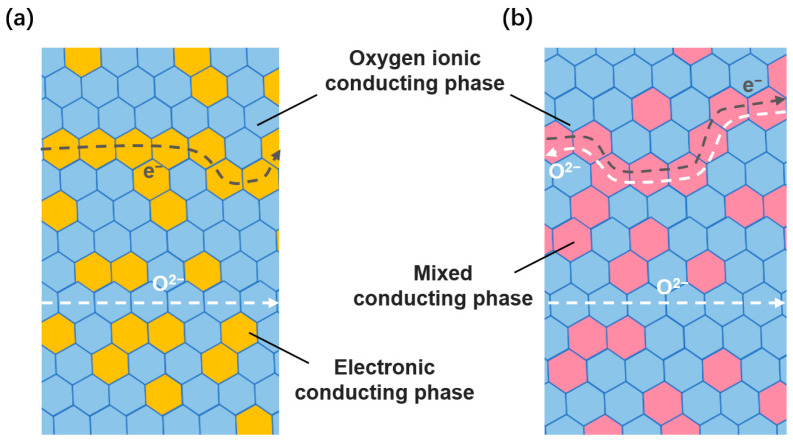
Permeation routes for dual–phase membranes consisting of (**a**) an oxygen ionic conducting and electronic conducting phase, and (**b**) an oxygen ionic conducting and mixed conducting phase.

A mixed conducting material with both high ionic conductivity and well–matched electronic conductivity is desirable but challenging to achieve in a single–component material. To overcome this challenge, Mazanec et al. [50] proposed a dual–phase membrane concept for methane conversion. The earliest dual–phase membrane consisted of a metal phase, such as Ag or Pt, and a fluorite phase, such as YSZ (as shown in Figure 4a). The former was used for electronic conduction, while the latter was used for oxygen ion conduction, enabling the omission of external circuits. In ideal dual–phase membranes, the ionic conductor is the continuous phase, and the electronic conductor is uniformly dispersed throughout the membrane network. The concept of dual–phase membranes provides additional opportunities in material selection, but also presents challenges in terms of fabrication methods.

In a dual–phase membrane, the electronic conductor phase is used to compensate for the low conductivity of the ionic conductor phase (Figure 4b). Perovskite is a good candidate for the electronic conductor phase due to its relatively low cost and compatibility with fluorite–type oxides. The combination of these two phases allows for simultaneous oxygen ionic and electronic transport in the membrane, extending the oxygen surface exchange reaction to the overall dual–phase membrane surface. This increases the surface exchange reaction rate and bulk diffusion for oxygen permeation, resulting in higher oxygen permeability. Therefore, the total oxygen conductivity of ceramic dual–phase membranes is larger than that of metal–ceramic dual–phase membranes, which further contributes to the higher permeability of ceramic dual–phase membranes [51,52].

Dual–phase membranes are generally more stable than single–phase perovskite membranes in the presence of CO_2_. This is because dual–phase membranes have a fluorite–type oxide phase that is predominantly oxygen ionic conducting and is stable in CO_2_–containing environments. Fang et al. [53,54] prepared a dual–phase membrane with a predominantly ionic conducting fluorite–type MIEC oxide (Ce_0.85_Gd_0.1_Cu_0.02_O_2−δ_(CGCO)) and a predominantly electronic conducting perovskite–type MIEC oxide (La_0.6_Ca_0.4_FeO_3−δ_(LCF)). With pure CO_2_ sweeping, a maximum oxygen permeation flux of 0.70 mLcm^−2^min^−1^ was obtained with a 0.5 mm thick membrane at 950 °C. It is worth mentioning that the membrane is stable in the presence of CO_2_, even at low temperatures (800 °C) during long–term operation, which is a characteristic not possessed by a single–phase perovskite membrane.

The flux of different typical oxygen–permeable membranes is summarized in Table 1.

### 3.3. Hydrogen–Permeable Membranes

In addition to their oxygen ion conductivity and mixed ionic and electronic conductivity, perovskite materials can also exhibit proton conductivity, making them useful for hydrogen–permeable membrane applications. For example, SrCeO_3_ doped with 5% Ce replaced by Yb shows protonic conduction in a hydrogen–containing atmosphere at high temperatures [68,69]. The presence of oxygen vacancies, which are created by doping, is believed to play a critical role in the formation and transport of protons in perovskite materials. Ln_6_WO_12_ is another promising material for hydrogen–permeable membranes. Ln_6_WO_12_ (Ln = La, Pr, Nd, Sm, Eu, and Gd) belongs to the family of double perovskite oxides, which have shown potential for various applications due to their unique electronic and ionic transport properties [70,71,72,73]. In particular, the oxygen vacancy concentration in Ln_6_WO_12_ can be significantly increased by doping of niobium and molybdenum, which enhances proton conduction at high temperatures in a hydrogen–containing atmosphere. This makes it a promising candidate for hydrogen–permeable membranes. Moreover, Ln_6_WO_12_ is also stable in CO_2_–containing atmospheres, which makes it an even more attractive option for industrial applications.

**Figure 5:**
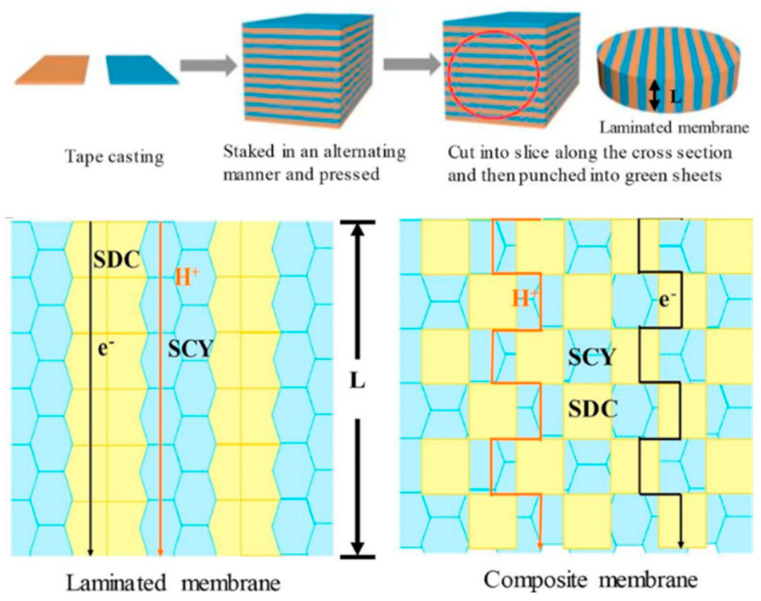
Schematic diagram of the laminated dual–phase membrane. Blue represents the SCY phase, and yellow represents the SDC phase. Reprinted with permission from Ref. [76]. Copyright 2019 Elsevier.

The interfacial resistance is the resistance to mass transfer across the interface between the two phases, which can limit the overall permeation rate of the membrane. In one study, a dual–phase membrane was prepared by mixing different amounts of Ni in the BaCe_1−x_Y_x_O_3_ ceramic phase [74]. The addition of nickel affected the surface properties of the membrane, but the interfacial resistance between the two phases was found to be much higher than the bulk resistance. This suggests that reducing the interfacial resistance between the two phases of a dual–phase membrane is important for improving its performance in hydrogen permeation [75]. A novel dual–phase SrCe_0.9_Y_0.1_O_3_–Ce_0.8_Sm_0.2_O_2_ (SCY–SDC)–laminated membrane was prepared, which consisted of alternating films of SCY and SDC phases (as shown in Figure 5) [76]. This laminated membrane was found to exhibit higher hydrogen (H_2_) flux than a conventional SCY–SDC dual–phase membrane, due to the shortened bulk diffusion distance for protons and electrons. The alternating films of the two phases allowed for more efficient proton and electron transport between the two phases, reducing the interfacial resistance and improving the overall hydrogen permeation rate. This approach of using laminated dual–phase membranes could have potential applications in fuel cells, where efficient proton and electron transport is critical for optimal performance. Further research is needed to optimize the composition and microstructure of the laminated membrane, as well as to evaluate its long–term stability and durability.

The flux of different typical hydrogen–permeable membranes is summarized in Table 2.

## 4. Membrane Architecture

**Figure 6:**
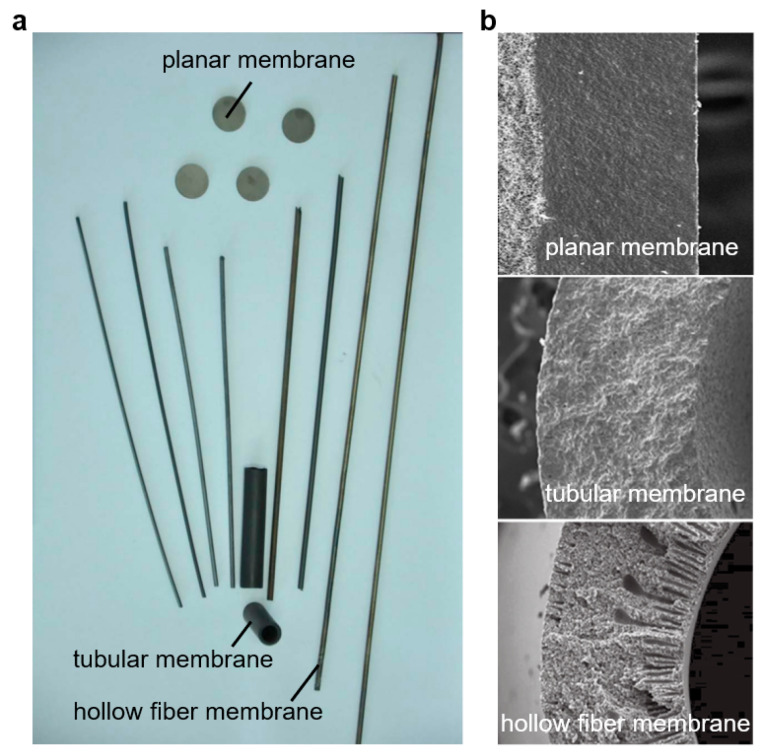
(**a**) The photographs and (**b**) the cross–sectional SEM images of the planar membranes, tubular membranes and hollow–fiber membranes. Reprinted with permission from Ref. [30]. Copyright 2013 China Science Press.

The architecture of a catalytic membrane reactor is an important factor that can influence its performance, and it can be considered from different standpoints. One important aspect of the architecture is the membrane configuration. There are several types of membrane configurations that can be used in CMRs, including planar, tubular, and hollow-fiber membranes (Figure 6) [30]. Another important aspect of the architecture is the membrane symmetry. Membranes can be symmetrical or asymmetrical. Asymmetrical membranes can be achieved by having a single dense layer with no porous support layers, or by having porous support layers on both sides of the dense layer, whereas an asymmetrical membrane typically has a gradient in pore size across the membrane thickness, and it has a dense layer on one side and a porous support layer on the other side.

The thickness of a membrane plays an important role in determining its mass transfer resistance and overall performance. In general, reducing the thickness of the membrane can decrease the mass transfer resistance and improve the efficiency of separation or reaction processes. A thin and dense separation layer with a porous support layer (a supported membrane) is considered a promising configuration for achieving high selectivity and permeance. However, preparing a supported dense membrane with a thin separation layer and good interfacial bonding can be challenging. Thermal and chemical compatibility between the membrane and the support, as well as the formation of a fully dense and defect–free top layer, are critical factors that must be addressed.

Jin et al. [83] proposed a versatile co–sintering technique to prepare a crack–free supported membrane, which involves directly coating a thin membrane precursor on the surface of a green support and sintering the two layers together in one step. This technique has been widely used by many researchers to prepare both planar and tubular ceramic membranes [84,85,86]. A study proposed a novel supported membrane structure that includes a catalytic activation layer, a gastight layer, and a porous substrate with 34% open porosity. The catalytic activation layer has a thickness of 17 μm, the gastight layer is 70 μm thick, and the porous substrate is 830 μm thick. The oxygen flux achieved by this membrane is impressive, reaching 10.0 mL (STP) min^−1^ cm^−2^ at 900 °C, which is 3 to 10 times higher than other membranes under similar conditions [64]. This structure showcases the potential of supported ceramic membranes to achieve high fluxes and highlights the importance of proper membrane design and optimization.

A tubular membrane is a type of membrane that has a cylindrical shape. Tubular membranes have several features. For example, large surface area: it can be made with a large surface area, allowing for efficient separation of fluids; high packing density: it can be packed tightly together, allowing for a large number of membranes to be used in a small space; and easy sealing at high temperatures: it can be directly inserted into a high–temperature reactor and sealed at the cold ends outside the furnace. Other important features of tubular membranes are their scalability, and they can be bundled together to form membrane modules with a larger surface area, which increases their efficiency in separating [87].

Hollow–fiber ceramic membranes have attracted significant attention in the last decade due to their unique asymmetric structure, which is formed by a phase inversion spinning/sintering technique [88,89,90,91,92]. It has a similar structure to tubular membranes, with both being cylindrical in shape. Compared to conventional planar or tubular membranes, hollow–fiber ceramic membranes offer several advantages. Firstly, they have a much larger membrane area per unit volume, which makes them more efficient at separating gases or filtering water. Secondly, the thin dense layer in the center (or at one side) of the fiber reduces bulk diffusion resistance, allowing faster and more efficient transport through the membrane. Finally, the integrated porous layers on either side or both sides of the membrane create large gas–membrane interfaces, resulting in enhanced gas surface exchange rates.

**Figure 7:**
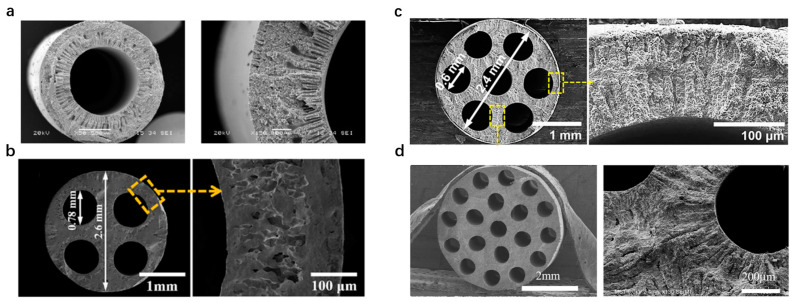
SEM images of (**a**) a single–channel hollow fiber with a dual–layer structure, reprinted with permission from Ref. [93], copyright 2010 Elsevier; and SEM images of different types of multi–channel hollow fibers: (**b**) 4–channel, reprinted with permission from Ref. [94], copyright 2015 John Wiley and Sons, (**c**) seven–channel (The yellow box are the morphology of the cross section of membranes), reprinted with permission from Ref. [95], copyright 2017 John Wiley and Sons, and (**d**) 19–channel. reprinted with permission from Ref. [96], copyright 2020 Elsevier.

The current research direction in hollow–fiber membranes is focused on developing membranes with enhanced properties that can meet the specific requirements of industrial applications. One approach is to develop multilayered hollow–fiber membranes that combine different materials with complementary properties. Wu et al. [93] developed a highly compact multifunctional hollow–fiber membrane reactor with a unique dual–layer structure for methane conversion using a single–step co–extrusion and co–sintering technique (Figure 7a). The membrane consists of a thin outer oxygen separation layer of approximately 75 μm and a porous inner support layer of a Ni–based catalytic substrate. The inner layer turns into a highly porous structure and becomes catalytically active for methane conversion after reduction. This membrane reactor has a high surface area/volume ratio and a great adhesion between the separation layer and the catalytic layer.

While improving the mechanical strength of hollow–fiber membranes is an important research direction, multi–channel hollow–fiber membranes consist of multiple channels or lumens within a single hollow fiber, which can provide higher mechanical strength compared to conventional single–channel hollow fibers. This design for oxygen–permeable membranes was proposed by Jin et al. [65,95,96]. A Ba_0.5_Sr_0.5_Co_0.8_Fe_0.2_O_3−δ_ (BSCF) four–channel hollow–fiber membrane was successfully fabricated (Figure 7b). Its breaking load and oxygen flux were several times those of conventional single–channel hollow–fiber membranes [65]. The high mechanical strength of the four–channel hollow–fiber membrane was helpful in overcoming some drawbacks of single–channel hollow–fiber membranes and ensuring the proper performance of the reaction process [94]. Subsequently, the researchers also proposed developing more complex hollow–fiber structures with even more channels and higher mechanical strength. The breaking loads of the BSCF seven– and 19–channel hollow–fiber membranes are significantly higher than those of single–channel hollow fibers, with the seven–channel membrane having a breaking load of 17 times [95] and the 19–channel membrane having a breaking load of 60 times [96] that of a single–channel hollow fiber (Figure 7c,d). This demonstrates the potential of multi–channel hollow–fiber membranes to provide improved mechanical strength and durability, making them a promising approach for enhancing the performance and reliability of membrane technology in various industrial applications.

## 5. Functions of the Membrane in CMR

**Figure 8:**
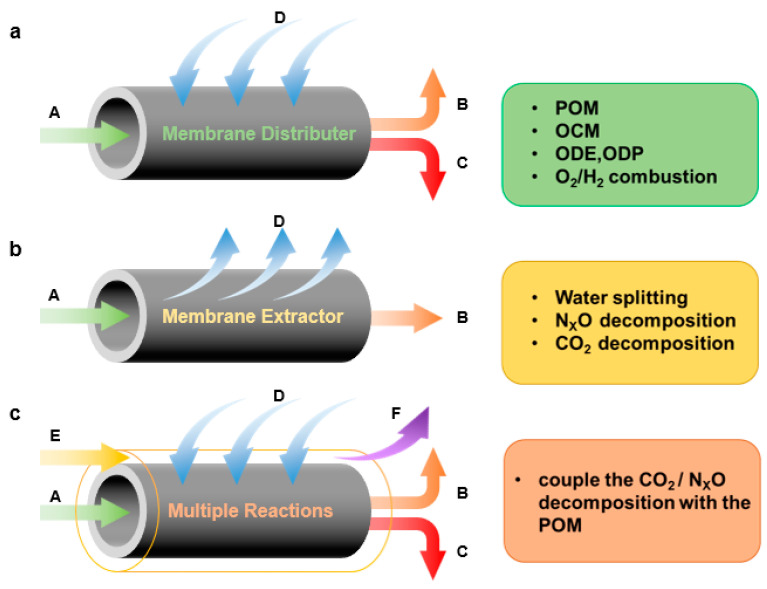
Basic functions of dense perovskite oxygen–permeable membranes in CMRs: (**a**) distribution of reactants; (**b**) preferential removal of products (**c**) coupling of multiple reactions. Where A is the reactant on one side of the membrane, B and C are the reaction products on that side. D is the reactant or product permeable through the membrane. E is the reactant on the other side of the membrane. F is the decomposition product of E.

In a dense ion–conducting membrane reactor, the membrane plays a crucial role in keeping oxidants and reductants separate, as well as selectively separating one of the substances. The specific functions of the membrane can be roughly divided into the following three categories (Figure 8).

Distribution of reactants: In some oxidation reactions, direct mixing of reactants can reduce reaction selectivity or increase explosion risk. In such cases, a membrane can act as a reactant distributor, increasing selectivity, minimizing risk, and utilizing the heat generated by exothermic reactions to heat reactants. As shown in Figure 8a, the reactant D was distributed by the membrane and reacted with reactant A to generate products B and C at one side of the membrane. This type of membrane reactor can be used for consecutive reactions or parallel reactions. Typical examples include partial oxidation of methane [97,98,99,100,101,102], oxidative coupling [26], and oxidative dehydrogenation of hydrocarbons [103,104]. By controlling the addition of reactant, the consecutive reaction can be controlled in the middle step or the desired reaction in the parallel reaction can be carried out preferentially, thus improving the yield of the target product. Typically, MIEC membranes are used for oxygen distribution, while MPEC membranes are used for hydrogen distribution.

Removal of products: In equilibrium–limited reactions, removing the products can shift the chemical equilibrium and increase the single–pass conversion. This can be achieved by using a membrane to selectively remove one of the products. As shown in Figure 8b, where A is a reactant and B and D are products, removing D via the membrane process shifts the equilibrium towards the desired direction. Mixed conducting membranes can be used to selectively remove oxygen or hydrogen from the reaction mixture, depending on the specific reaction and application. For example, perovskite membranes have been used for the CO_2_ thermal decomposition [105], where the removal of oxygen can enhance the selectivity towards CO production, other reactions were also reported, such as water spitting [106] and NO_x_ decomposition [107]. Similarly, perovskite membranes have been used for hydrogenation and dehydrogenation reactions, where the removal of hydrogen can improve the yield of the target product. Example reactions are water gas shift reaction [108], ammonia decomposition reaction [109], alkane to olefin reaction [110], methane steam reforming reaction [111] and methane to aromatic reaction [112,113].

Coupling of multiple reactions: A new function has been developed that combines the functions of dense ceramic membranes for reactant distribution and product removal on both sides of the membrane. In this design, reactant E decomposes to D and F on one side of the membrane, with D permeating through the membrane to react with A and produce B and C on the other side (Figure 8c). For example, a membrane reactor can be used to couple the decomposition reactions (such as CO_2_, H_2_O and NO_X_ decomposition) with the preferential reaction (such as partial oxidation of methane [98,99,114,115] or hydrogen oxidation [116]) to consume the oxygen or hydrogen and increase the conversion of decomposition. Particularly, a membrane reactor can be used to couple exothermic and endothermic reactions to achieve an autothermic condition. In this case, the heat generated by the exothermic reaction is used to drive the endothermic reaction, resulting in a self–sustaining process [117,118].

Reactions that can be performed in ion–conducting ceramic membrane reactors are summarized in Table 3.

## 6. Applications of CMRs

### 6.1. Partial Oxidation of Methane

Partial oxidation of methane is a chemical reaction in which methane is reacted with a limited amount of oxygen to produce a mixture of carbon monoxide and hydrogen gas, known as syngas. The produced syngas can then be converted into liquid fuel (methanol) and hydrogen using Fischer–Tropsch synthesis and water gas shift reactions, respectively [10,99]. The partial oxidation of methane is an exothermic reaction that releases heat as the reaction proceeds. If the heat generated by the reaction is not effectively dissipated, the temperature of the reaction mixture can increase rapidly, leading to a phenomenon known as temperature runaway [98]. Temperature runaway can cause several problems, including reduced product selectivity, increased energy consumption, and potential damage to the reactor. To prevent temperature runaway in the partial oxidation of methane, one approach is to control the oxygen flow rate and the reaction temperature to maintain a steady–state condition.

Perovskite oxygen–permeable membranes have been developed for use in partial oxidation reactions, including the partial oxidation of methane. These materials have the ability to selectively transport oxygen ions across the membrane, which can be used to regulate the rate of oxygen supply to the reaction zone and help prevent temperature runaway. The use of mixed conducting membranes enables the separation of oxygen from air as the oxidant without introducing nitrogen into the reaction compartment, simplifying the purification of product streams and reducing syngas production costs [38]. Efforts have been made to improve the oxygen permeability and chemical stability of the membrane materials, resulting in the development of materials such as Ba–Sr–Co–Fe by Shao et al. [119] and Ba–Co Fe–Zr by Tong et al. [120].

The stability of perovskite materials in methane partial oxidation reactions is a significant challenge that limits their further application. The harsh operating conditions of methane partial oxidation can cause perovskite materials to undergo structural changes, which can lead to a decrease in oxygen permeability [121]. One potential solution to this problem is the use of K_2_NiF_4_–type materials or dual–phase materials. Dong et al. proposed a self–catalytic membrane reactor for POM reactions based on an asymmetric MIEC membrane [122,123]. La_2_NiO_4+δ_, which has catalytic activity for CH_4_ reforming, is used as both the porous support and the catalyst precursor. In the initial reaction stage of the POM reaction, CH_4_ reacts with La_2_NiO_4+δ_ to form CO and H_2_, and simultaneously, part of La_2_NiO_4+δ_ on the surface of the support is reduced to nickel metal and La_2_O_3_.

Zhu et al. [24] investigated oxygen permeation and partial oxidation of methane (POM) in 75 wt% Ce_0.85_Sm_0.15_O_1.925_-25 wt% Sm_0.6_Sr_0.4_FeO_3−δ_ (SDC–SSF) dual–phase composite membrane reactors. The dual–phase membrane consists of a fluorite–type oxide ionic conductor (SDC) for oxide ion transport and a perovskite–type mixed conductor (SSF) for both oxide ion and electron transport. The permeation flux remained constant during the investigated period (>500 h) once a steady state was reached. The dual–phase membrane reactors achieved successful long–term operation of POM with methane conversion and CO selectivity >98% when pure methane was used as the feed. Characterization revealed good structural stability for the dual–phase membrane even after long–term operation under syngas production conditions. Jin’s group modified a single hollow–fiber membrane into a multi–channel hollow–fiber membrane to increase its mechanical strength and build a more robust membrane reactor [94,95,96]. The reactor’s capacity increases with more membrane channels. Table 4 provides further performance comparisons.

### 6.2. Oxidative Coupling of Methane

Methane oxidative coupling (OCM) to produce ethylene or ethane is a promising process for converting methane into more valuable C2 products. However, the oxidation rate of desired C2 products is higher than that of methane, leading to a limited yield of products [35]. In addition, the formation of CO_x_ in the gas phase reaction and the 25% equilibrium conversion rate of OCM reaction are also problematic.

To address these issues, researchers have focused on using dense oxygen–permeable ceramic membranes for oxidative methane coupling. One promising approach involves the use of mixed conducting oxides, such as La_0.2_Sr_0.8_CoO_3−δ_, Ba_0.5_Sr_0.5_Co_0.8_Fe_0.2_O_3−δ_, and BaCe_0.8_Gd_0.2_O_3−δ_, which have shown good OCM catalytic properties with C2 yields ranging from 14% to 16% and C2 selectivity from 40% to over 80% [34]. The BaCe_0.8_Gd_0.2_O_3−δ_–based membrane/catalyst system in particular has demonstrated excellent carbon resistance at low O_2_/CH_4_ ratios and high and stable selectivity to C2+ products.

Compared to perovskite and fluoride materials, other materials such as Bi_1.5_Y_0.3_Sm_0.2_O_3−δ_ (BYS) have also exhibited good performance in methane coupling reactions. A BYS membrane reactor achieved a 35% yield of C2 product, which is higher than the commercial consideration threshold of C2 at around 30% [133]. The BYS material is highly oxygen permeable and catalytically active for OCM, with good chemical and mechanical stability under OCM conditions. When BYS is deposited as the OCM catalyst, a micro–structured La_0.6_Sr_0.4_Co_0.2_Fe_0.8_O_3−δ_ (LSCF) hollow–fiber membrane can achieve higher oxygen permeation rate and methane conversion, provided the BYS particles are small and uniformly dispersed [134]. Furthermore, the distributed feed of oxygen in the form of dissociated or ionized oxygen can react with methane on the surface of the membrane or the catalyst, minimizing the formation of CO_x_ and increasing the selectivity of C2 hydrocarbons.

### 6.3. Oxidative Dehydrogenation of Hydrocarbons

The demand for olefins, particularly ethylene and propylene, is expected to increase significantly. One potential route to produce these compounds is through the oxidative dehydrogenation (ODH) of alkanes. This method offers advantages such as being exothermic and having the presence of oxygen limit coke formation [135]. However, the yields of ethylene or propylene in the existing catalytic reaction are too low for commercial applications. One of the main challenges is the fact that the desired products are more readily oxidized to CO_x_. By using dense ceramic membranes as oxygen distributors, the contact of the catalyst with oxygen can be minimized, leading to improved target product yield.

**Figure 9:**
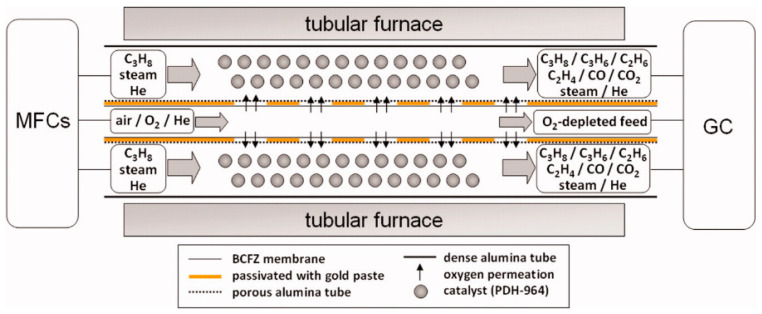
Schematic drawing of the reactor set–up and an incorporated multi–step BCFZ hollow fiber with five active zones for oxidative dehydrogenation of propane. Except for the five oxygen permeable zones, the membrane is coated with gold. Each permeation zone is 2 cm long. The dehydrogenation catalyst was dispersed outside the hollow fiber. Reprinted with permission from Ref. [104]. Copyright 2010 John Wiley and Sons.

Many researchers have confirmed this general expectation [102,103,136]. Wang et al. [102] used a BaCo_x_Fe_y_Zr_z_O_3−δ_ (BCFZ) hollow fiber for oxidative dehydrogenation of the ethane to ethylene reaction. However, the best ethylene selectivity of 64% was lower than the 79% obtained with the BCFZ disk membrane. This difference was mainly attributed to the different contact time on the two membrane configurations. To further study this problem, researchers divided a hollow fiber into several segments along the axial direction [103,104]. As shown in Figure 9, oxygen permeation zones and inert zones coated with gold paste are arranged alternately. The highest propene selectivity of 75% was observed at a propane conversion of 26% and 625 °C (88% total olefin selectivity), while the best propene yield of 36% was achieved at 725 °C (69% total olefin selectivity).

The oxidative dehydrogenation of the propane reaction was investigated using a catalytic BaBi_0.05_Co_0.8_Nb_0.15_O_3−δ_ perovskite hollow–fiber membrane reactor. The results showed that high C_3_H_6_ yield (~50%) and selectivity (~74%) can be achieved, which are more than double the yield and selectivity obtained in a conventional co–feed mode reactor [137]. In a study using parametric modeling, the oxidative dehydrogenation of propane was investigated in a La_0.6_Sr_0.4_Co_0.2_Fe_0.8_O_3−δ_ (LSCF) hollow–fiber membrane reactor. The oxygen permeation flux was found to be dependent on temperature, gas atmosphere, and membrane thickness. The highest projected flux of 10.2 mL (STP) cm^−2^min^−1^ was achieved through a 0.2 mm thick fiber at 1100 °C. Simulation results showed that the optimum C_3_H_6_ selectivity of 91% was achieved at 1000 °C when C_3_H_8_ conversion reached 58% [138]. However, the high operating temperature and oxygen flux may cause excessive oxidation in practical experiments, which can ultimately reduce performance [137].

Table 5 provides additional performance comparisons of OCM and ODH reactions.

### 6.4. Water Splitting Reaction

Water is an inexpensive industrial material, so it has been considered for producing hydrogen by splitting it into oxygen and hydrogen at high temperatures ($H_{2}O(g)=H_{2}+\frac{1}{2}O_{2}$). However, the equilibrium constant of the water dissociation reaction is very small, resulting in only 0.1% of hydrogen and 0.042% of oxygen being generated at 1600 °C. In comparison with the steam electrolysis process, the application of oxygen or hydrogen–permeable mixed conduction membranes can disrupt the balance and shift it towards the product side, without the need for extra power or circuit [145,146,147]. For instance, a 0.13 mm thick dual–phase membrane, composed of 60% Gadolinia–Doped Ceria (GDC) and 40% metal by volume, was used for directly separating hydrogen from water splitting [145]. The production rate of hydrogen is 0.6 cm^3^cm^−2^·min^−1^ at 900 °C.

### 6.5. Alkane to Olefin Reaction

In the non–oxidative coupling of methane to C2 products, hydrogen removal via a mixed proton–electronic membrane reactor can significantly improve the C2 yield [148]. This reactor continuously removes hydrogen from the dehydrogenation reaction of methane, thus shifting the reaction equilibrium towards the product side. In contrast to the oxygen distributor membrane reactor, the hydrogen removal membrane reactor can restrict CO_x_ by–products, since no oxygen is introduced, and thus the selectivity is likely higher. Further research on applying mixed electronic protonic conducting ceramic membranes in direct coupling of methane into C2 products has been focused on preparing ultrathin separation layers and achieving a large ratio of membrane area to volume of membrane module, as well as improving the catalytic properties of the membranes through catalyst loading by various methods [149]. So far, the preparation of ultrathin ceramic membranes remains a significant challenge in membrane fabrication technology.

In a product removal reactor, dense ceramic membranes are typically employed to eliminate hydrogen generated during the decomposition process. In traditional chemical processes such as methane partial oxidation (${CH}_{4}+\frac{1}{2}O_{2}=CO+2H_{2}$), steam reforming of natural gas (${CH}_{4}+H_{2}O=CO+3H_{2}$), water gas shift reaction ($CO+H_{2}O=CO_{2}+H_{2}$), or gasification of heavy carbonaceous materials, dense membranes can be used to separate hydrogen from the resulting high–temperature hydrogen mixtures. By recovering hydrogen from high–temperature and high–pressure streams without cooling or depressurizing, the system’s efficiency and performance can be enhanced. Recovered hydrogen is 100% pure, thereby reducing the cost of producing pure hydrogen.

### 6.6. Coupling of Reactions

The coupling of two or more reactions in one membrane reactor has been proposed as a sustainable process for the future. This approach allows for the integration of different reaction steps into a single system, and for simultaneous execution of multiple reactions with different requirements for reactant addition and product removal, which can lead to improved reaction efficiency, reduced energy consumption, and lower greenhouse gas emissions. Some thermodynamically unfavorable reactions (such as CO_2_ decomposition or water splitting) may be promoted by introducing an active reaction (such as the POM) on the other side of the membrane (Figure 8).

#### 6.6.1. CO_2_ Decomposition

One example of such a reactor is the thermal decomposition of carbon dioxide (TDCD) to CO and O_2_. This reaction is limited by thermodynamic equilibrium and is challenging to realize in a conventional fixed–bed reactor. However, with a membrane reactor, this problem can be solved [150]. In the work of Jin et al. [150,151,152], an MIEC membrane reactor was used to integrate the TDCD and POM processes into a single unit. TDCD occurred on one side of the membrane, and the POM occurred simultaneously on the other side. This process produced H_2_ and CO through the reaction of methane with oxygen, which permeated through the membrane from CO_2_ decomposition, over supported transition metal catalysts. This work indicates that MIEC membrane reactors may play a crucial role in effectively utilizing fossil energy and greenhouse gases. However, the MIEC membrane reactor’s stability was found to be no longer than 60 h, and the membrane broke significantly due to erosion by CO_2_ and reducing atmospheres. Future research may focus on developing MIEC materials with high stability in both CO_2_ and reducing atmospheres. It is worth noting that the proposed catalytic process can also be extended to the decomposition of other oxygen–containing molecules (such as NO_x_ and H_2_O) and the oxidation of light alkanes (such as ethane and propane).

#### 6.6.2. NO_x_ Decomposition

Nitrogen oxide (NO_x_) is an important atmospheric pollutant that has attracted significant attention. Direct catalytic decomposition of NO_x_ to N_2_ and O_2_ is an attractive and economical route for NO_x_ reduction. Jiang et al. [153] proposed an interesting process that combined the decomposition of nitrous oxide (N_2_O) with the oxidation steam reforming of methane in a BCFZ hollow–fiber membrane reactor. N_2_O was catalytically decomposed on the membrane surface of the core side to produce N_2_ and surface oxygen species (O*), which were removed as oxygen ions (O^2−^) through the membrane. Meanwhile, methane and water were fed to the permeate side of the membrane to react with the permeated oxygen and produce syngas. By using the MIEC membrane for in situ oxygen removal, the N_2_O decomposition was promoted, overcoming the equilibrium limitation. At 875 °C, a nearly 100% N_2_O conversion was achieved when the N_2_O concentration was in the range of 5% to 50% in the feed gas. The same group further studied a similar process in which NO was used instead of N_2_O as the feed gas in the membrane reactor [154]. An almost 100% NO conversion and 95% N_2_ yield were achieved at 875 °C, even with 3% O_2_ coexisting in the feed.

#### 6.6.3. Water Splitting

Direct thermochemical water splitting is highly endothermic, so coupling it with an exothermic reaction is preferable. Jiang et al. [155] studied this concept first. They combined water splitting with POM in a BCFZ hollow–fiber membrane reactor with a Ni–based catalyst on the membrane surface. At high temperature, water decomposed into hydrogen and oxygen on the membrane surface of the core side. Oxygen permeated from the core to the shell side of the hollow fiber, where it was consumed by the POM reaction to form syngas. The advantage of this process is the continuous production of pure hydrogen as well as syngas. Furthermore, the highly endothermic property of water splitting can be partially compensated by the exothermic POM reaction. Cao et al. [156] achieved the combination of thermal water splitting with oxidative coupling of methane (OCM) to produce ethane and ethylene using an asymmetric Ba_0.5_Sr_0.5_Co_0.8_Fe_0.2_O_3−δ_ disc membrane with a 2wt% Mn–5wt% Na_2_WO_4_ catalyst. Oxygen generated from thermal water splitting was transported through the dense BSCF membrane and consumed during the coupling of methane. At 950 °C, approximately 9% of the injected H2O was converted to hydrogen, with a production rate of about 3.3 mL cm^−2^min^−1^ with 26% CH_4_ conversion and 6.5% C2 selectivity.

**Figure 10:**
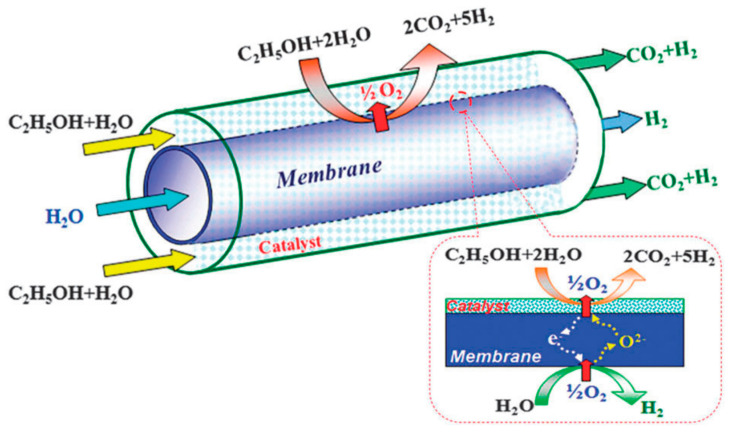
Schematic diagram of the water splitting and oxidative steam reforming coupling membrane reactor. Reprinted with permission from Ref. [118]. Copyright 2012 Royal Society of Chemistry.

Another innovative approach to obtain purified hydrogen is the combination of water splitting with oxidative steam reforming of ethanol, developed by Zhu et al. [118]. They used a tubular SrCo_0.4_Fe_0.5_Zr_0.1_O_3−δ_ membrane reactor packed with Ni catalyst to achieve a highly effective and sustainable route for H_2_ production. Water splitting occurred at the tube side of the membrane, while the oxidative steam reforming of ethanol occurred at the shell side. Ethanol and water reacted with oxygen, which permeated through the membrane upon water dissociation, to produce H_2_ and CO_2_ over supported transition metal catalysts. These two reactions were coupled to the opposite sides of the oxygen–permeable membrane. At 750 °C, the hydrogen production rates on the ethanol side and the water side were 6.8 and 1.8 mLcm^−2^min^−1^, respectively (Figure 10).

**Figure 11:**
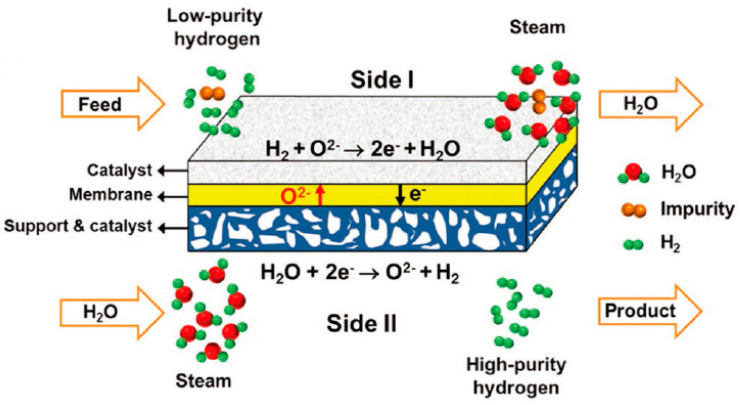
Oxygen–permeable membrane reactor for hydrogen purification. Reprinted with permission from Ref. [116]. Copyright 2016 John Wiley and Sons.

A membrane reactor can also be used for gas purification. Li et al. proposed using MIEC oxygen–permeable membranes to obtain high–purity hydrogen via the water splitting reaction [116]. To achieve this, low–purity hydrogen was fed to one side of the membrane (side I) and steam was fed to the other side (side II) (Figure 11). Oxygen produced from water splitting on side II permeates through the membrane, driven by an oxygen chemical potential gradient across the membrane, to react with the low–purity hydrogen on side I. High–purity hydrogen is then acquired after condensation and drying. A high hydrogen separation rate of up to 16.3 mL cm^−2^ min^−1^ was achieved on an asymmetric dual–phase membrane at 900 °C.

## 7. Challenges

Dense ceramic membrane reactors have shown significant potential for various energy and environment–related applications, such as hydrogen production, methane conversion, CO_2_ capture and utilization, and harmful gas treatment. However, despite their potential, the commercialization of dense ceramic membrane reactors has been slow, and there are several challenges that need to be addressed before their widespread use can be realized.

**Figure 12:**
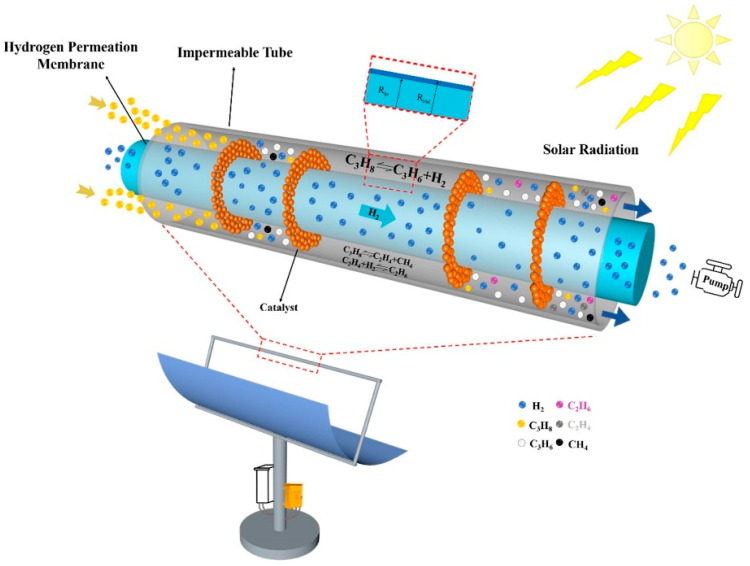
Conceptual solar–driven propane dehydrogenation system integrated with HPM reactor. Reprinted with permission from Ref. [165]. Copyright 2021 Elsevier.

One of the major challenges with perovskite membrane reactors is their high energy demand, which is required to maintain the operating temperature, generally above 700 °C. The use of electric heating results in high energy consumption and a large carbon emission load. Solar thermo processes have been proposed as a promising solution to this challenge. For example, the current dish–type solar thermal system can achieve a thermal–to–electric conversion efficiency of 75%; however, the average photoelectric conversion efficiency of current commercial photovoltaic components is only about 15% [157]. Additionally, using solar thermal energy to drive the hydrogen production process can store low–energy–density solar energy in high–energy–density and green hydrogen. Thus, solar thermal–driven hydrogen production processes have been widely implemented worldwide, including solar–driven chemical chain hydrogen production [158], steam reforming gasification [159], and thermal cracking gasification hydrogen production [160]. Research on using concentrated solar energy with CMRs is currently a hot and frontier topic, including high–temperature water decomposition hydrogen production [161], carbon dioxide decomposition [162], and non–oxidative methane dehydroaromatization [163]. For instance, Tou et al. [164] used a solar concentrating system to heat a catalytic membrane reactor, generating 3500 suns of irradiance and heating the reactor to 1600 °C, with a carbon dioxide decomposition rate greater than 1.44 μmol·cm^−2^·min^−1^. Liang et al. [165] used a Ce_0.9_Pr_0.1_O_2−δ_–Pr_0.6_Sr_0.4_FeO_3−δ_ dual–phase oxygen transport membrane reactor with a solar oven (as shown in Figure 12). The reactor produced synthesis gas with a production rate of 1.3 mL min^−1^cm^−2^, H_2_O conversion above 1.7%, and CO_2_ conversion above 4.2% (930 °C, H_2_O/CO_2_ feed ratio of 5:1).

**Figure 13:**
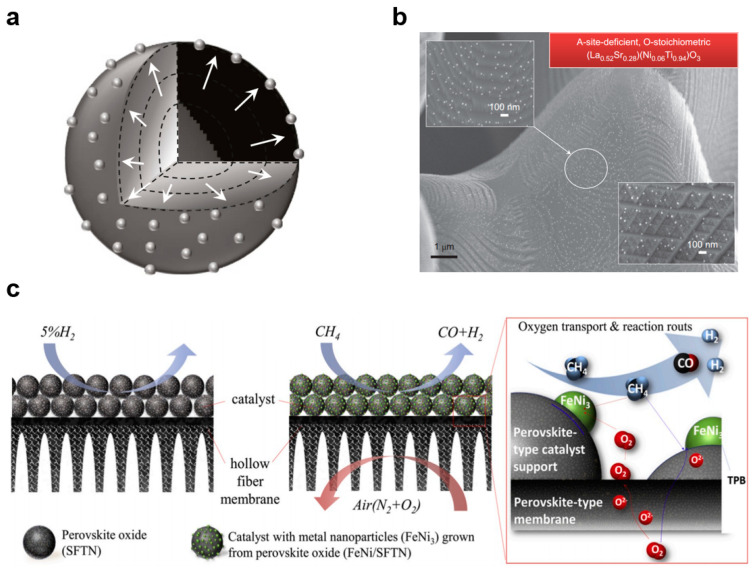
(**a**) Schematic illustration of particle exsolution on perovskite oxides, reprinted with permission from Ref. [171], copyright 2016 Springer Nature, (**b**) the SEM images of exsolved nanoparticles, reprinted with permission from Ref. [168], copyright 2013 Springer Nature, (**c**) schematic diagrams of the homologous design of CMRs, reprinted with permission from Ref. [172], copyright 2020 Elsevier.

Another challenge is the need for efficient and selective catalysts that can work effectively with ceramic membranes. The choice of catalyst can significantly impact the efficiency and selectivity of the reactions, and the cost and stability of the catalysts can also be a significant factor. Metal catalysts (iron, nickel, copper, palladium, etc.) are widely used in ceramic membrane reactors [118,151]. People usually disperse metal particles on a substrate to maximize the accessible specific surface area of the catalyst and thus improve the catalytic activity. However, the metal particles loaded on the surface of the substrate by conventional preparation methods, including impregnation, co–precipitation and deposition, often tend to sinter and grow after high–temperature annealing (>700 °C), resulting in a severe loss of their catalytic activity. Balancing the two main requirements of stability and activity has proven to be a difficult goal. In recent years, perovskite oxides have attracted much attention as cost–effective catalysts. Previous studies have demonstrated that the B–site active transition metals in perovskite oxides have the potential to grow in situ into metal nanoparticles and anchor on the surface after treatment with a reducing atmosphere (Figure 13a,b) [166,167,168]. These surface–anchored nanoparticles exhibit high stability against agglomeration and poisoning, as well as superior catalytic activity across multiple areas of application [169,170,171]. Jin et al. [172] first proposed employing homogenous Sr_0.9_(Fe_0.81_Ta_0.09_Ni_0.1_)O_3−δ_ perovskite catalysts with well–anchored FeNi_3_ nanoparticles in a ceramic membrane reactor for methane partial oxidation reaction (Figure 13c). A great leap forward in the performance of catalytic membrane reactors has been achieved. The methane conversion, carbon monoxide selectivity and hydrogen selectivity reached 98%, 97% and 98%, respectively. The membrane severs in order to distribute oxygen, control the oxygen partial pressure and effectively prevent the dissolution of metal particles during oxidation reactions.

**Figure 14:**
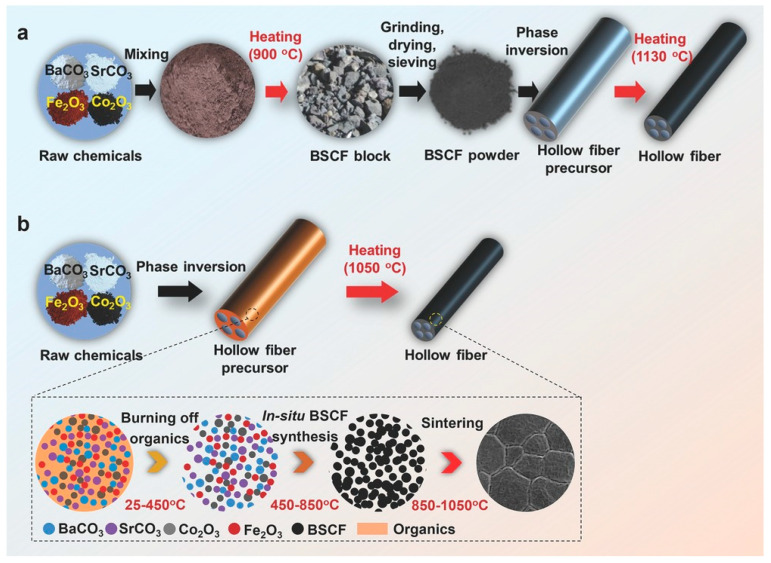
Schematic of perovskite hollow–fiber membranes fabrication approaches. (**a**) Traditional multi–step approach. (**b**) One–step thermal processing (OSTP) approach and perovskite hollow–fiber membranes evolution mechanism during single thermal processing. Reprinted with permission from Ref. [173]. Copyright 2017 John Wiley and Sons.

Furthermore, there are also challenges related to scaling up the membrane fabrication from the laboratory to the industrial scale. Hollow–fiber membranes have been demonstrated to be preferable for practical applications. Typically, the fabrication process for perovskite hollow–fiber membranes involves multiple steps, including high–temperature synthesis of perovskite powder, phase inversion for spinning the hollow fiber precursor, and high–temperature sintering. These processes are not only labor, energy, and time consuming, they are also environmentally unfriendly and it is difficult to precisely control the cation stoichiometry of the perovskite oxides. The development of more efficient and sustainable methods for preparing perovskite hollow–fiber membranes could greatly facilitate the scaling up of membrane reactor technology for industrial use. Zhu et al. developed a one–step thermal processing approach for the fabrication of perovskite hollow fibers [173]. Unlike traditional methods, which require multiple steps and high temperatures, this approach directly introduces raw chemicals (such as oxides or carbonates) into the phase inversion process, and then converts them in situ into perovskite oxides during a single thermal processing step. This approach also successfully avoids the reaction of perovskite oxide with solvent or non–solvent used in HF fabrication and achieved a controlled stoichiometry. For example, Ba_0.5_Sr_0.5_Co_0.8_Fe_0.2_O_3−δ_ hollow–fiber membranes have been successfully fabricated using this approach, and exhibit high oxygen permeation flux values that exceed the desired commercial targets (Figure 14).

## 8. Conclusions and Outlook

In conclusion, the utilization of ion–conducting ceramic membranes, specifically MIEC and MPEC membranes, in membrane reactors offers a promising opportunity for sustainable chemical production. These membranes possess the ability for selective gas separation at elevated temperatures, ultimately reducing by–product formation and facilitating the utilization of thermal effects to promote chemical reactions. The integration of CMRs provides a plethora of benefits, including partial oxidation of methane to syngas, steam reforming for hydrogen production, and thermal decomposition of carbon dioxide. In addition to the production of high–value chemicals and hydrogen from natural gas, the utilization of biomass is a crucial area for the future development of CMRs. Biomass fermentation offers a significant advantage as a renewable energy source, enabling the production of biofuels such as bioethanol and biomethane. One distinguishing feature of biomass–based systems is their CO_2_ neutrality. During the biofuel production process, the CO_2_ emitted is absorbed by biomass growth, resulting in a closed carbon cycle and a reduced carbon footprint. The application of a membrane reactor for the oxidation steam reforming of bioethanol or biomethane shows great potential for efficient hydrogen production. Additionally, an intriguing area for future research involves integrating the oxidation steam reforming of biofuels with water splitting in a dense ceramic membrane reactor. This integration would enable high hydrogen productivity on both sides of the membrane, further enhancing the overall system efficiency.

Despite their significant potential in various reactions, the commercialization of dense ceramic membrane reactors remains sluggish due to several challenges that require addressing before their widespread use can be realized. One significant challenge is reducing the energy required for these processes by integrating renewable energy sources such as solar thermal energy. Furthermore, the development of efficient catalytic structures with stable nanostructures at high temperatures is critical to the success of these processes. Lastly, large–scale membrane fabrication strategies must be developed to enable the commercialization of these membranes. Overall, this review provides a comprehensive overview of the various types of membrane reactors, their principles, advantages, disadvantages, and key issues. Additionally, the paper discusses the configuration and design of catalytic membrane reactors and provides insights into the challenges of scaling up membrane reactors from experimental stages to practical applications. The future of dense ceramic catalytic membrane reactors is promising, and further research and development can lead to significant advancements in the field of sustainable chemical production.

## Figures and Tables

**Table 1:** Comparison of oxygen permeation fluxes of membranes with various materials.

Material	Membrane Type	Thickness (mm)	Feed Gas	Sweep Gas	Temperature (°C)	Oxygen Flux **(**$mL\cdot {min}^{-1}{cm}^{-2}$**)**	Stability	Ref.
SrCo_0.8_Fe_0.2_O_3−δ_	disk	1.0	Air	He	877	3.1	/	[44]
SrCo_0.4_Fe_0.6_O_3−δ_	disk	1.0	Air	He	877	2.4	/	[44]
La_0.2_Sr_0.8_Co_0.4_Fe_0.6_O_3−δ_	disk	1.0	Air	He	877	0.6	/	[44]
La_0.6_Sr_0.4_CoO_3−δ_	disk	1.5	Air	He	860	1.0	/	[55]
La_0.6_Sr_0.4_Co_0.8_Fe_0.2_O_3−δ_	disk	1.5	Air	He	860	0.62	/	[55]
La_0.6_Sr_0.4_Co_0.8_Mn_0.2_O_3−δ_	disk	1.5	Air	He	860	0.50	/	[55]
La_0.6_Sr_0.4_Co_0.8_Ni_0.2_O_3−δ_	disk	1.5	Air	He	860	1.44	/	[55]
La_0.2_Sr_0.8_Co_0.2_Fe_0.8_O_3−δ_	disk	2	Air	He	850	0.32	/	[56]
SrCo_0.8_Fe_0.2_O_3−δ_	disk	1	Air	Inert gas	900	4.24	/	[57]
Ba_0.5_Sr_0.5_Co_0.8_Fe_0.2_O_3−δ_	hollow fiber	0.22	Air	He	900	3.80	/	[58]
BaCe_0.15_Fe_0.85_O_3−δ_	disk	1	Air	He	900	0.418	/	[59]
BaCe_0.15_Fe_0.85_O_3−δ_	disk	1.4	Air	He	940	0.47	1 h in 5% H_2_ + Ar	[60]
		0.56				0.81		
BaBi_0.3_Co_0.2_Fe_0.5_O_3−δ_	disk	1.5	Air	He	900	0.5	/	[61]
		0.66				0.85		
SrCo_0.9_Nb_0.1_O_3−δ_	disk	1	21% oxygen−He	Ar	900	4.24	200 days	[62]
SrCo_0.9_Ta_0.1_O_3−δ_	disk	0.65	Air	He	900	1.83	520 h	[63]
Ba_0.5_Sr_0.5_Co_0.8_Fe_0.2_O_3−δ_	disk	0.9 (0.07 dense)	Air	Ar	900	10.0	/	[64]
SrCo_0.8_Fe_0.2_O_3−δ_ +_0.5_%wt Nb_2_O_5_	4−channel hollow fiber	0.2 (0.01 dense)	Air	He	900	1.6	500 h	[65]
SrFe_0.8_Nb_0.2_O_3−δ_	disk	1	Air	CO_2_	900	0.25	210 h	[2]
SrFe_0.9_Ta_0.1_O_3−δ_	disk	1	Air	CO_2_	900	0.30	130 h	[66]
SrFe_0.9_Ta_0.1_O_3−δ_	4−channel hollow fiber	0.25	Air	CO_2_	900	1.15	130 h	[66]
BaCo_0.7_Fe_0.22_Nb_0.08_O_3−δ_	4−channel hollow fiber	0.13	Air	He	900	8.1	/	[67]
		0.13			650	1.15	300 h	[67]

**Table 2:** Comparison of hydrogen–permeable fluxes of membranes with various materials.

Material	Membrane Type	Thickness (mm)	Feed Gas	Sweep Gas	Temperature (°C)	Oxygen Flux **(**$mL\cdot {min}^{-1}{cm}^{-2}$**)**	Stability	Ref.
Ba(Ce_0.7_Zr_0.1_Y_0.1_Yb_0.1_)_0.95_Ni_0.05_O_3−δ_	disk	0.45	10% H_2_−N_2_	Ar	800	0.42	Stable at 700 °C for 200 h	[77]
		0.6				0.24		
SrCe_0.9_Y_0.1_O_3−δ_	disk	0.8	40% H_2_−N_2_	Ar	900	0.10	/	[78]
BaCe_0.9_Y_0.1_O_3−δ_	disk	0.8	40% H_2_−N_2_	Ar	900	0.75	/	[78]
Ba_0.5_Sr_0.5_Ce_0.9_Y_0.1_O_3−δ_	disk	0.8	40% H_2_−N_2_	Ar	900	1.60	/	[78]
LaCe_0.9_Y_0.1_O_3−δ_	disk	0.8	40% H_2_−N_2_	Ar	900	0.02	/	[78]
Pd modified−BaCe_0.95_Tb_0.05_O_3−δ_	hollow fiber	0.26	50% H_2_−Ne	N_2_	900	0.27	/	[79]
BaCe_0.85_Tb_0.05_Co_0.1_O_3−δ_	hollow fiber	0.75	50% H_2_−Ne	N_2_	1000	0.38	/	[80]
BaCe_0.95_Nd_0.05_O_3−δ_	disk	0.7	80% H_2_+He+H_2_O	Ar	925	0.026	/	[81]
BaZr_0.1_Ce_0.2_Y_0.7_O_3−δ_	disk		50% H_2_−N_2_	Ar	900	0.018	Stable at 850 °C for 2 h	[82]
BaZr_0.3_Ce_0.6_Y_0.1_Zn_0.05_ O_3−δ_	disk		50% H_2_−N_2_	Ar	900	0.012	Stable at 850 °C for 4 h	[82]

**Table 3:** Reactions that can be performed in ion–conducting ceramic membrane reactors.

Reaction	Reaction Equation	$∆H^{0}$ **(** kJ·mol ^−1^ **)**	Membrane Transport	Temperature **(**°C**)**
Partial oxidation of methane	${CH}_{4}+\frac{1}{2}O_{2}\to CO+2H_{2}$	−36	O^2−^/e−	850–900
Oxidative coupling of methane	$2{CH}_{4}+O_{2}\to C_{2}H_{4}+{2H}_{2}O$ ${2CH}_{4}+\frac{1}{2}O_{2}\to C_{2}H_{6}+H_{2}O$	−177 −282	O^2−^/e^−^	688–955
Oxidative dehydrogenation of hydrocarbons	$C_{2}H_{6}+{\frac{1}{2}O}_{2}\to C_{2}H_{4}+H_{2}O$ $C_{3}H_{8}+{\frac{1}{2}O}_{2}\to C_{3}H_{6}+H_{2}O$	−105	O^2−^/e^−^	700–850
Water splitting reaction	$H_{2}O\to H_{2}+{\frac{1}{2}O}_{2}$	−242	O^2−^/e^−^ H^+^/e^−^	900
Alkane to olefin reaction	$2{CH}_{4}\to C_{2}H_{6}+H_{2}$ $2{CH}_{4}\to C_{2}H_{4}+H_{2}$	71.1 57.2	H^+^/e^−^	750–900
Carbon dioxide decomposition	$2{CO}_{2}\to 2CO+O_{2}$	552	O^2−^/e^−^	>900
Steam reforming	${CH}_{4}+H_{2}O\to CO+3H_{2}$ ${C_{2}H}_{5}OH+H_{2}O\to 2CO+4H_{2}$	210 256	O^2−^/e^−^	800
Dry reforming	${CH}_{4}+{CO}_{2}\to 2CO+2H_{2}$	260.5	O^2−^/e^−^	700–1000
Dehydroaromation of methane	${CH}_{4}\to C_{6}H_{6}+2H_{2}$ ${CH}_{4}+O_{2}\to C_{6}H_{6}+2H_{2}O$	531 −1846	H^+^/e^−^ O^2−^/e^−^	700–750

**Table 4:** Different membrane reactors for POM reaction.

Material	Membrane Type	Thickness (mm)	Feed Gas	Reaction Gas	Temp. (°C)	Oxygen Flux **(**$mL\;{min}^{-1}{cm}^{-2}$**)**	CH_4_ Conversion (%)	CO Selectivity (%)	Stability	Reference
Ba_0.5_Sr_0.5_Co_0.8_Fe_0.2_O_3−δ_	disk	1.50	Air	50% CH_4_+He	875	11.50	98.2	96.0	500 h	[39]
BaCo_0.4_Fe_0.4_Zr_0.2_O_3−δ_	disk	1.0	Air	50% CH_4_+He	850	5.7	97.5	98.5	2200 h	[120]
Ba_0.5_Sr_0.5_Zn_0.2_Fe_0.8_O_3−δ_	disk	1.25	Air	50% CH_4_+He	950	2.55	60.0	98.3	65 h	[124]
BaCe_0.1_Co_0.4_Fe_0.5_O_3−δ_	disk	1.0	Air	CH_4_	875	8.9	>97.0	>97.0	1000 h	[125]
75wt.% Ce _0.85_Sm _0.15_O_1.925_ –25 wt.% Sm_0.6_Sr_0.4_FeO_3−δ_	disk	0.6	Air	CH_4_	950	4.2	>98.0	>98.0	650 h	[24]
La_0.4_Ba_0.6_Fe_1−x_Zn_x_O_3−δ_	disk	0.5	Air	17.5% CH_4_+He	950	7.80	100	72	500 h	[126]
				100% CH_4_	950	11.8	55	100	180 h	[126]
Ba_0.9_Co_0.7_Fe_0.2_Nb_0.1_O_3−δ_	disk	1	Air	30% CH_4_+He	875	7.10	93.4	94.5	400 h	[127]
Ba_0.5_Sr_0.5_Co_0.8_Fe_0.1_N_i0.1_O_3−δ_	disk	Not	Air	50% CH_4_+He	850	12.00	98.0	97.5	120 h	[100]
SrCo_0.8_Fe_0.2Nb_O_3−δ_ (Ba_0.3_Sr_0.7_Fe_0.9_Mo_0.1_O_3−δ_)	disk	1.0	Air	13% CH_4_+He	850	13.0	80.0	98.84	1500 h	[128]
BaCo_0.7_Fe_0.2_Ta_0.1_O_3−δ_	hollow fiber	0.23	Air	55.8% CH_4_+He	875	20.0	96	99	83 h	[129]
(Pr_0.9_La_0.1_)_2_ (Ni_0.74_Cu_0.21_Ga_0.05_)O_4_+_δ_	hollow fiber	0.19	Air	20.3% CH_4_+He	900	10.5	97	99.5	140 h	[101]
Ba_0.9_Co_0.7_Fe_0.2_Nb_0.1_O_3−δ_	disk	0.12	Air	30% CH_4_+Ar	875	16.00	96.66	78.7	100 h	[130]
SrFe_0.8_Nb_0.2_O_3−δ_	4−channel hollow fiber	0.08	Air	17% CH_4_+He	900	19.2	94.6	99	120 h	[94]
SrCo_0.8_Fe_0.1_Ga_0.1_O_3−δ_	hollow fiber	0.21	Air	20% CH_4_+He	800	4.14	100	33	220 h	[131]
BaBi_0.05_Co_0.8_Nb_0.15_O_3−δ_	hollow fiber	0.13	Air	50% CH_4_+He	730	15.05	80	85	5 h	[132]

**Table 5:** Performance comparisons of OCM and ODH reactions.

Material	Membrane Type	Thickness (mm)	Feed Gas	Reaction Gas	Temp. (°C)	Oxygen Flux **(**$mL\;{min}^{-1}{cm}^{-2}$**)**	Conversion (%)	Selectivity (%)	Yield (%)	Reaction & Stability	Ref.
BaCe_0_._8_Gd_0_._2_O_3−δ_	tube	0.7	Air	3% CH_4_+He	780	0.1	26 (CH_4_)	62 (C_2_)	>16 (C_2_)	OCM	[35]
Ba_0.5_Sr_0.5_Co_0.8_Fe_0.2_O_3−δ_	tube	1.7	Air	10% CH_4_+He	850	1.50	5 (CH_4_)	62 (C_2_)	15 (C_2_)	OCM	[139]
Ba_0.5_Sr_0.5_Co_0.8_Fe_0.2_O_3−δ_	disk	1.0	Air	34% CH_4_+He	900	7.0	25 (CH_4_)	70 (C_2_)	18 (C_2_)	OCM	[25]
Ba_0.5_Sr_0.5_Co_0.8_Fe_0.2_O_3−δ_	disk	1.0	Air	11% CH_4_+He	900	2.00	23 (CH_4_)	63 (C_2_)	15 (C_2_)	OCM	[25]
Ba_0.5_Ce_0.4_Gd_0.1_Co_0.8_Fe_0.2_O_3−δ_	tube	3.0	Air	50% CH_4_+He	850	1.40	51.6 (CH_4_)	67.4 (C_2_)	34.7 (C_2_)	OCM	[140]
La_0.6_Sr_0.4_Co_0.2_Fe_0.8_O_3−δ_	hollow fiber	0.25	Air	75% CH_4_+Ar	900	8.73	49 (CH_4_)	79.5 (C_2_)	39 (C_2_)	OCM (18 h)	[134]
BaCe_0.8_Gd_0.2_O_3−δ_	disk	0.5	Air	95% CH_4_+He	880	2.1	/	80 (C_2_)	/	OCM (25 h)	[141]
Ni–La_0.8_Sr_0.2_Ga_0.8_Mg_0.2_O_3−δ_	disk	0.5	Air	95% CH_4_+He	880	0.9	/	30 (C_2_)	/	OCM (25 h)	[141]
Ba_0.5_Sr_0.5_Co_0.8_Fe_0.2_O_3−δ_	planar	1.0	Air	10% C_2_H_6_+He	800	1.75	84 (C_2_H_6_)	80 (C_2_)	/	ODH (100 h)	[142]
BaCo_x_Fe_y_Zr_z_O_3−δ_	hollow fiber	0.1	Air	10% C_2_H_6_+He	800	1.15	89.6 (C_2_H_6_)	39.9 (C_2_)	/	ODH	[143]
BaCo_x_Fe_y_Zr_z_O_3−δ_	hollow fiber	0.14	Air	10% C_3_H_8_+ 20% H_2_O+He	675	/	48 (C_3_H_8_)	59 (C_3_H_6_)	28 (C_2_)	ODH	[104]
Ce_0.9_Gd_0.1_O_2−δ_–BaFe_0.9_Mg_0.05_Ce_0.05_O_3−δ_	disk	0.5	Air	5% C_2_H_6_+He	725	/	74 (C_2_H_6_)	82 (C_2_H_4_)	63 (C_2_)	ODH (200 h)	[144]
BaBi_0.05_Co_0.8_Nb_0.15_O_3−δ_	hollow fiber	0.12	Air	C_3_H_8_	650	/	62–75 (C_3_H_8_)	68–74 (C_3_H_6_)	46–51 (C_3_H_6_)	ODH (50 h)	[137]

## Data Availability

No new data were created or analyzed in this study. Data sharing is not applicable to this article.

## References

[1] Dong X.L., Jin W.Q., Xu N.P., Li K. (2011). Dense ceramic catalytic membranes and membrane reactors for energy and environmental applications. Chem. Commun..

[2] Yi J., Schroeder M., Martin M. (2013). CO_2_–Tolerant and Cobalt–Free SrFe_0.8_Nb_0.2_O_3−δ_ Perovskite Membrane for Oxygen Separation. Chem. Mater..

[3] Ning H., Wei Z., Wei G., Sijie X., Chi Z., Xuan Z., Jan F., Shaomin L. (2021). Novel oxygen permeable hollow fiber perovskite membrane with surface wrinkles. Sep. Purif. Technol..

[4] Zhu Y., Lei J., Liu J., Tan J., Zhang G., Liu Z., Jin W. (2023). Fabrication of CO_2_–tolerant SrFe_0.8_Nb_0.2_O_3−δ_/SrCo_0.9_Nb_0.1_O_3−δ_ dual–layer 7–channel hollow fiber membrane by co–spinning and one–step thermal process. J. Membr. Sci..

[5] Zhang Z.C., Ning K., Xu Z., Zheng Q.K., Tan J.K., Liu Z.K., Wu Z.T., Zhang G.R., Jin W.Q. (2021). Highly efficient preparation of Ce_0.8_Sm_0.2_O_2−δ_ SrCo_0.9_Nb_0.1_O_3−δ_ dual–phase four–channel hollow fiber membrane via one–step thermal processing approach. J. Membr. Sci..

[6] Wang L., Chen Y., Wang G., Li Y., Bai M., Hall D., Dou R. (2018). A case study of the effect of Ni substitution on the sintering behaviours of Ba_0.5_Sr_0.5_Co_0.8_Fe_0.2_O_3−δ_ oxygen transport membranes. Adv. Appl. Ceram..

[7] Song J., Hei Y., Li C., Yang N., Meng B., Tan X., Sunarso J., Liu S. (2022). Dehydrogenation Coupling of Methane Using Catalyst–Loaded Proton–Conducting Perovskite Hollow Fiber Membranes. Membranes.

[8] Fairuzov D., Gerzeliev I., Maximov A., Naranov E. (2021). Catalytic Dehydrogenation of Ethane: A Mini Review of Recent Advances and Perspective of Chemical Looping Technology. Catalysts.

[9] Liu Y., Yuan S.Z., Xie K. (2021). Conversion of Methane to Ethylene with BaCe_0.9_Y_0.1_Co_x_O_3−δ_ Hydrogen Permeation Membrane. Chin. J. Struct. Chem..

[10] Mohanty U.S., Ali M., Azhar M.R., Al–Yaseri A., Keshavarz A., Iglauer S. (2021). Current advances in syngas (CO+H_2_) production through bi–reforming of methane using various catalysts: A review. Int. J. Hydrogen Energy.

[11] Schmeda–Lopez D.R., Nunes E.H.M., Vasconcelos D., Vasconcelos W.L., Meulenberg W.A., Diniz da Costa J.C. (2018). The Neck to Particle Ratio Effect on the Mechanical and Morphological Sintering Features of Porous Stainless Steel (SS) Hollow Fibers. Adv. Eng. Mater..

[12] Gui T., Zhang F., Li Y.Q., Cui X., Wu X.W., Zhu M.H., Hu N., Chen X.S., Kita H., Kondo M. (2019). Scale–up of NaA zeolite membranes using reusable stainless steel tubes for dehydration in an industrial plant. J. Membr. Sci..

[13] Shin J.H., Yu H.J., An H., Lee A.S., Hwang S.S., Lee S.Y., Lee J.S. (2019). Rigid double–stranded siloxane–induced high–flux carbon molecular sieve hollow fiber membranes for CO_2_/CH_4_ separation. J. Membr. Sci..

[14] Wang D.Q., Gao Y.F., Gao S.J., Huang H.K., Min F., Li Y.X., Seeger S., Jin J., Chu Z.L. (2023). Antifouling superhydrophilic porous glass membrane based on sulfobetaine prepared by thiol–ene click chemistry for high–efficiency oil/ water separation. J. Membr. Sci..

[15] Liu Y.Y., Tan X.Y., Li K. (2006). Mixed conducting ceramics for catalytic membrane processing. Catal. Rev. Sci. Eng..

[16] Bouwmeester H.J.M., Burggraaf A.J. (1996). Dense ceramic membranes for oxygen separation. Membr. Sci. Technol..

[17] Kharton V.V., Yaremchenko A.A., Kovalevsky A.V., Viskup A.P., Naumovich E.N., Kerko P.F. (1999). Perovskite–type oxides for high–temperature oxygen separation membranes. J. Membr. Sci..

[18] Al S., Zhang G. (2018). The Role of Metal Catalyst on Water Permeation and Stability of BaCe_0.8_Y_0.2_O_3−δ_. J. Electrochem. Sci. Technol..

[19] Zhang Q.-Y., Han J.-J., Huang Y., Chen Y., Yan X., Lang W.-Z. (2020). Effect of Ba non–stoichiometry in Ba_1−x_Zr_0.1_Ce_0.7_Y_0.2_O_3−δ_ on its structure defect, sinterability and hydrogen permeability. Ceram. Int..

[20] Tao Z., Yan L., Qiao J., Wang B., Zhang L., Zhang J. (2015). A review of advanced proton–conducting materials for hydrogen separation. Prog. Mater. Sci..

[21] Yaremchenko A., Kharton V., Valente A., Veniaminov S., Belyaev V., Sobyanin V., Marques F. (2007). Methane oxidation over mixed–conducting SrFe(Al)O_3−δ_–SrAl_2_O_4_ composite. Phys. Chem. Chem. Phys..

[22] Tan X., Li K. (2009). Design of mixed conducting ceramic membranes/reactors for the partial oxidation of methane to syngas. AIChE J..

[23] Dong X., Zhang G., Liu Z., Zhong Z., Jin W., Xu N. (2009). CO_2_–tolerant mixed conducting oxide for catalytic membrane reactor. J. Membr. Sci..

[24] Zhu X., Li Q., He Y., Cong Y., Yang W. (2010). Oxygen permeation and partial oxidation of methane in dual–phase membrane reactors. J. Membr. Sci..

[25] Olivier L., Haag S., Mirodatos C., van Veen A.C. (2009). Oxidative coupling of methane using catalyst modified dense perovskite membrane reactors. Catal. Today.

[26] Czuprat O., Schiestel T., Voss H., Caro J. (2010). Oxidative Coupling of Methane in a BCFZ Perovskite Hollow Fiber Membrane Reactor. Ind. Eng. Chem. Res..

[27] Wei Y.Y., Yang W.S., Caro J., Wang H.H. (2013). Dense ceramic oxygen permeable membranes and catalytic membrane reactors. Chem. Eng. J..

[28] Hashim S.S., Somalu M.R., Loh K.S., Liu S.M., Zhou W., Sunarso J. (2018). Perovskite–based proton conducting membranes for hydrogen separation: A review. Int. J. Hydrogen Energy.

[29] Zhu X., Yang W. (2019). Microstructural and Interfacial Designs of Oxygen–Permeable Membranes for Oxygen Separation and Reaction–Separation Coupling. Adv. Mater..

[30] Jin W.X., Nanping (2013). Mixed–Conducting Oxygen–Permeation Materials (Book).

[31] Zhang K., Sunarso J., Shao Z., Zhou W., Sun C., Wang S., Liu S. (2011). Research progress and materials selection guidelines on mixed conducting perovskite–type ceramic membranes for oxygen production. RSC Adv..

[32] Tejuca L.G., Fierro J.L.G., Tascón J.M.D. (1989). Structure and Reactivity of Perovskite–Type Oxides. Adv. Catal..

[33] Kharton V., Viskup A., Naumovich E., Lapchuk N. (1997). Mixed electronic and ionic conductivity of LaCo(M)O_3_ (M=Ga, Cr, Fe or Ni): I. Oxygen transport in perovskites LaCoO_3_–LaGaO_3_. Solid State Ion..

[34] Lin Y., Zeng Y. (1996). Catalytic properties of oxygen semipermeable perovskite–type ceramic membrane materials for oxidative coupling of methane. J. Catal..

[35] Lu Y., Dixon A.G., Moser W.R., Ma Y.H., Balachandran U. (2000). Oxygen–permeable dense membrane reactor for the oxidative coupling of methane. J. Membr. Sci..

[36] Jin W., Gu X., Li S., Huang P., Xu N., Shi J. (2000). Experimental and simulation study on a catalyst packed tubular dense membrane reactor for partial oxidation of methane to syngas. Chem. Eng. Sci..

[37] Jin W., Li S., Huang P., Xu N., Shi J., Lin Y. (2000). Tubular lanthanum cobaltite perovskite–type membrane reactors for partial oxidation of methane to syngas. J. Membr. Sci..

[38] Shao Z., Dong H., Xiong G., Cong Y., Yang W. (2001). Performance of a mixed–conducting ceramic membrane reactor with high oxygen permeability for methane conversion. J. Membr. Sci..

[39] Shao Z., Xiong G., Dong H., Yang W., Lin L. (2001). Synthesis, oxygen permeation study and membrane performance of a Ba_0. 5_Sr_0. 5_Co_0. 8_Fe_0. 2_O_3−δ_ oxygen–permeable dense ceramic reactor for partial oxidation of methane to syngas. Sep. Purif. Technol..

[40] Itoh N., Sanchez M.A., Xu W.C., Haraya K., Hongo M. (1993). Application of a membrane reactor system to thermal–decomposition of CO_2_. J. Membr. Sci..

[41] Han N., Wei Q., Zhang S.G., Yang N.T., Liu S.M. (2019). Rational design via tailoring Mo content in La_2_Ni_1−x_Mo_x_O_4+delta_ to improve oxygen permeation properties in CO_2_ atmosphere. J. Alloys Compd..

[42] Halat D.M., Dunstan M.T., Gaultois M.W., Britto S., Grey C.P. (2018). Study of Defect Chemistry in the System La_2−x_Sr_x_NiO_4+delta_ by O–17 Solid–State NMR Spectroscopy and Ni K–Edge XANES. Chem. Mater..

[43] Cheng J., Zhang S., Meng B., Ding J., Tan X. (2018). Preparation and the superior oxygen permeability of a new CO_2_–resistant Ruddlesden–Popper composite oxide Pr_2_Ni_0.9_Mo_0.1_O_4+δ_. J. Alloys Compd..

[44] Teraoka Y., Zhang H.M., Furukawa S., Yamazoe N. (1985). Oxygen permeation through perovskite–type oxides. Chem. Lett..

[45] Akin F., Lin J.Y. (2004). Oxygen permeation through oxygen ionic or mixed–conducting ceramic membranes with chemical reactions. J. Membr. Sci..

[46] Lin Y.S. (2001). Microporous and dense inorganic membranes: Current status and prospective. Sep. Purif. Technol..

[47] Shao Z.P., Yang W.S., Cong Y., Dong H., Tong J.H., Xiong G.X. (2000). Investigation of the permeation behavior and stability of a Ba_0.5_Sr_0.5_Co_0.8_Fe_0.2_O_3−delta_ oxygen membrane. J. Membr. Sci..

[48] Kharton V., Naumovich E., Kovalevsky A., Viskup A., Figueiredo F., Bashmakov I., Marques F. (2000). Mixed electronic and ionic conductivity of LaCo(M)O_3_ (M=Ga, Cr, Fe or Ni): IV. Effect of preparation method on oxygen transport in LaCoO_3−δ_. Solid State Ion..

[49] Li S., Jin W., Huang P., Xu N., Shi J., Hu M.Z.-C., Payzant E.A., Ma Y.H. (1999). Perovskite–related ZrO_2_–doped SrCo_0.4_Fe_0.6_O_3−δ_ membrane for oxygen permeation. AIChE J..

[50] Mazanec T.J., Cable T.L., Frye J.G., Kliewer W.R. (1999). Solid multi–component membranes, electrochemical reactor components, electrochemical reactors and use of membranes, reactor components, and reactor for oxidation reactions. J. Membr. Sci..

[51] Wang H.H., Yang W.S., Cong Y., Zhu X.F., Lin Y.S. (2003). Structure and oxygen permeability of a dual–phase membrane. J. Membr. Sci..

[52] Kharton V., Kovalevsky A., Viskup A., Shaula A., Figueiredo F., Naumovich E., Marques F. (2003). Oxygen transport in Ce_0.8_Gd_0.2_O_2−δ_–based composite membranes. Solid State Ion..

[53] Fang W., Liang F., Cao Z., Steinbach F., Feldhoff A. (2015). A Mixed Ionic and Electronic Conducting Dual–Phase Membrane with High Oxygen Permeability. Angew. Chem. Int. Ed..

[54] Fang W., Steinbach F., Chen C., Feldhoff A. (2015). An approach to enhance the CO_2_ tolerance of fluorite–perovskite dual–phase oxygen–transporting membrane. Chem. Mater..

[55] Yasutake T., Takashi N., Noboru Y. (1988). Effect of Cation Substitution on the Oxygen Semipermeability of Perovskite–type Oxides. Chem. Lett..

[56] Li S., Jin W., Xu N., Shi J. (1999). Synthesis and oxygen permeation properties of La_0.2_Sr_0.8_Co_0.2_Fe_0.8_O_3−δ_ membranes. Solid State Ion..

[57] Tan L., Yang L., Gu X., Jin W., Zhang L., Xu N. (2004). Influence of the size of doping ion on phase stability and oxygen permeability of SrCo_0.8_Fe_0.2_O_3−δ_ oxide. J. Membr. Sci..

[58] Liu S., Gavalas G. (2005). Oxygen selective ceramic hollow fiber membranes. J. Membr. Sci..

[59] Zhu X., Wang H., Yang W. (2004). Novel cobalt–free oxygen permeable membrane. Chem. Commun..

[60] Zhu X., Cong Y., Yang W. (2006). Oxygen permeability and structural stability of BaCe_0.15_Fe_0.85_O_3−δ_ membranes. J. Membr. Sci..

[61] Shao Z., Xiong G., Cong Y., Yang W. (2000). Synthesis and oxygen permeation study of novel perovskite–type BaBi_x_Co_0.2_Fe_0 8−x_O_3−δ_ ceramic membranes. J. Membr. Sci..

[62] Nagai T., Ito W., Sakon T. (2007). Relationship between cation substitution and stability of perovskite structure in SrCoO_3−δ_−based mixed conductors. Solid State Ion..

[63] Huang L., Wei Y., Wang] H. (2011). Tantalum stabilized SrCoO_3−δ_ perovskite membrane for oxygen separation. J. Membr. Sci..

[64] Serra J.M., Lobera M.P., Schulze–Küppers F., Meulenberg] W.A. (2011). Ultrahigh oxygen permeation flux through supported Ba_0.5_Sr_0.5_Co_0.8_Fe_0.2_O_3−δ_ membranes. J. Membr. Sci..

[65] Zhu J., Dong Z., Liu Z., Zhang K., Zhang G., Jin W. (2014). Multichannel mixed–conducting hollow fiber membranes for oxygen separation. AIChE J..

[66] Zhu J., Guo S., Chu Z., Jin W. (2015). CO_2_–tolerant oxygen–permeable perovskite–type membranes with high permeability. J. Mater. Chem. A.

[67] Song Z., Zhang Z., Zhang G., Liu Z., Zhu J., Jin W. (2017). Effects of polymer binders on separation performance of the perovskite–type 4–bore hollow fiber membranes. Sep. Purif. Technol..

[68] Iwahara H., Esaka T., Uchida H., Maeda N. (1981). Proton conduction in sintered oxides and its application to steam electrolysis for hydrogen production. Solid State Ion..

[69] Iwahara H. (1996). Proton conducting ceramics and their applications. Solid State Ion..

[70] Escolástico S., Solís C., Scherb T., Schumacher G., Serra J.M. (2013). Hydrogen separation in La_5.5_WO_11.25–δ_ membranes. J. Membr. Sci..

[71] Chen T., Zhao H., Xie Z., Xu N., Lu Y. (2015). Oxygen permeability of Ce_0.8_Sm_0.2_O_2−δ_–LnBaCo_2_O_5+δ_ (Ln=La, Nd, Sm, and Y) dual–phase ceramic membranes. Ionics.

[72] Chen Y., Cheng S., Chen L., Wei Y., Ashman P.J., Wang H. (2016). Niobium and molybdenum co–doped La_5.5_WO_11.25−δ_ membrane with improved hydrogen permeability. J. Membr. Sci..

[73] Chen Y., Liu H., Zhuang L., Wei Y., Wang H. (2019). Hydrogen permeability through Nd_5.5_W_0.35_Mo_0.5_Nb_0.15_O_11.25−δ_ mixed protonic–electronic conducting membrane. J. Membr. Sci..

[74] Siriwardane R., Poston Jr J., Fisher E., Lee T., Dorris S., Balachandran U. (2000). Characterization of ceramic hydrogen separation membranes with varying nickel concentrations. Appl. Surf. Sci..

[75] Zhang G., Dorris S.E., Balachandran U., Liu M. (2003). Interfacial resistances of Ni–BCY mixed–conducting membranes for hydrogen separation. Solid State Ion..

[76] Meng B., Wang H.N., Cheng H.D., Wang X.B., Meng X.X., Sunarso J., Tan X.Y., Liu S.M. (2019). Hydrogen permeation performance of dual–phase protonic–electronic conducting ceramic membrane with regular and independent transport channels. Sep. Purif. Technol..

[77] Yang M., He F., Zhou C., Dong F., Yang G., Zhou W., Shao Z. (2021). New perovskite membrane with improved sintering and self–reconstructed surface for efficient hydrogen permeation. J. Membr. Sci..

[78] Heidari M., Safekordi A., Zamaniyan A., Ganji Babakhani E., Amanipour M. (2015). Comparison of microstructure and hydrogen permeability of perovskite type ACe_0.9_Y_0.1_O_3−δ_ (A is Sr, Ba, La, and BaSr) membranes. Int. J. Hydrogen Energy.

[79] Song J., Meng B., Tan X., Liu S. (2015). Surface–modified proton conducting perovskite hollow fibre membranes by Pd–coating for enhanced hydrogen permeation. Int. J. Hydrogen Energy.

[80] Song J., Li L., Tan X., Li K. (2013). BaCe_0.85_Tb_0.05_Co_0.1_O_3−δ_ perovskite hollow fibre membranes for hydrogen/oxygen permeation. Int. J. Hydrogen Energy.

[81] Cai M., Liu S., Efimov K., Caro J., Feldhoff A., Wang H. (2009). Preparation and hydrogen permeation of BaCe_0.95_Nd_0.05_O_3−δ_ membranes. J. Membr. Sci..

[82] Zhang K., Sunarso J., Pham G.H., Wang S., Liu S. (2014). External short circuit–assisted proton conducting ceramic membrane for H_2_ permeation. Ceram. Int..

[83] Jin W.Q., Li S.G., Huang P., Xu N.P., Shi J. (2001). Preparation of an asymmetric perovskite–type membrane and its oxygen permeability. J. Membr. Sci..

[84] Kovalevsky A., Kharton V., Maxim F., Shaula A., Frade J. (2006). Processing and characterization of La_0. 5_Sr_0. 5_FeO_3_–supported Sr_1−x_Fe (Al)O_3_–SrAl_2_O_4_ composite membranes. J. Membr. Sci..

[85] Feng J., Fan Y., Qi H., Xu N. (2007). Co–sintering synthesis of tubular bilayer α−alumina membrane. J. Membr. Sci..

[86] Watenabe K., Yuasa M., Kida T., Teraoka Y., Yamazoe N., Shimanoe K. (2010). High-Performance Oxygen-Permeable Membranes with an Asymmetric Structure Using Ba_0.95_La_0.05_FeO_3−δ_ Perovskite-Type Oxide. Adv. Mater..

[87] Li S.G., Jin W.Q., Huang P., Xu N.P., Shi J., Lin Y.S. (2000). Tubular lanthanum cobaltite perovskite type membrane for oxygen permeation. J. Membr. Sci..

[88] Tan X.Y., Liu S.M., Li K. (2001). Preparation and characterization of inorganic hollow fiber membranes. J. Membr. Sci..

[89] Khayet M. (2003). The effects of air gap length on the internal and external morphology of hollow fiber membranes. Chem. Eng. Sci..

[90] Pan X.L., Stroh N., Brunner H., Xiong G.X., Sheng S.S. (2003). Pd/ceramic hollow fibers for H_2_ separation. Sep. Purif. Technol..

[91] De Jong J., Benes N.E., Koops G.H., Wessling M. (2004). Towards single step production of multi–layer inorganic hollow fibers. J. Membr. Sci..

[92] Li D.F., Chung T.S., Rong W. (2004). Morphological aspects and structure control of dual–layer asymmetric hollow fiber membranes formed by a simultaneous co–extrusion approach. J. Membr. Sci..

[93] Wu Z., Wang B., Li K. (2010). A novel dual–layer ceramic hollow fibre membrane reactor for methane conversion. J. Membr. Sci..

[94] Zhu J.W., Guo S.B., Liu G.P., Liu Z.K., Zhang Z.C., Jin W.Q. (2015). A robust mixed–conducting multichannel hollow fiber membrane reactor. Aiche J..

[95] Zhu J.W., Wang T.L., Song Z., Liu Z.K., Zhang G.R., Jin W.Q. (2017). Enhancing oxygen permeation via multiple types of oxygen transport paths in hepta–bore perovskite hollow fibers. Aiche J..

[96] Wang T.L., Liu Z.K., Xu X.L., Zhu J.W., Zhang G.R., Jin W.Q. (2020). Insights into the design of nineteen–channel perovskite hollow fiber membrane and its oxygen transport behaviour. J. Membr. Sci..

[97] Tan X.Y., Thursfield A., Metcalfe I.S., Li K. (2009). Analysis of a perovskite ceramic hollow fibre membrane reactor for the partial oxidation of methane to syngas. Asia Pac. J. Chem. Eng..

[98] Kleinert A., Feldhoff A., Schiestel T., Caro J.G. (2006). Novel hollow fibre membrane reactor for the partial oxidation of methane. Catal. Today.

[99] Zhang S., Wang S., Jin Y., Song J., Meng X., Meng B., Yang N., Tan X., Zhu Z., Liu S. (2021). One stone two birds: Simultaneous realization of partial oxidation of methane to syngas and N_2_ purification via robust ceramic oxygen–permeable membrane reactors. Chem. Eng. J..

[100] Babakhani E.G., Towfighi J., Taheri Z., Pour A.N., Zekordi M., Taheri A. (2012). Partial oxidation of methane in Ba_0.__5_Sr_0.__5_Co_0.__8_Fe_0.__1_Ni_0.__1_O_3−__δ_ ceramic membrane reactor. J. Nat. Gas Chem..

[101] Wei Y., Liao Q., Li Z., Wang H., Feldhoff A., Caro J. (2014). Partial Oxidation of Methane in Hollow–Fiber Membrane Reactors Based on Alkaline–Earth Metal–Free CO_2_–Tolerant Oxide. Aiche J..

[102] Wang H., Tablet C., Schiestel T., Werth S., Caro J. (2006). Partial oxidation of methane to syngas in a perovskite hollow fiber membrane reactor. Catal. Commun..

[103] Czuprat O., Werth S., Schirrmeister S., Schiestel T., Caro J. (2009). Olefin production by a multistep oxidative dehydrogenation in a perovskite hollow-fiber membrane reactor. ChemCatChem.

[104] Czuprat O., Werth S., Caro J., Schiestel T. (2010). Oxidative dehydrogenation of propane in a perovskite membrane reactor with multi-step oxygen insertion. AIChE J..

[105] Kleiminger L., Li T., Li K., Kelsall G.H. (2014). CO_2_ splitting into CO and O_2_ in micro–tubular solid oxide electrolysers. Rsc. Adv..

[106] Tan X.Y., Capar G., Li K. (2005). Analysis of dissolved oxygen removal in hollow fibre membrane modules: Effect of water vapour. J. Membr. Sci..

[107] Wang Z., Li Z., Cui Y., Chen T., Hu J., Kawi S. (2019). Highly Efficient NO Decomposition via Dual–Functional Catalytic Perovskite Hollow Fiber Membrane Reactor Coupled with Partial Oxidation of Methane at Medium–Low Temperature. Environ. Sci. Technol..

[108] Wang M., Zhou Y., Tan X., Gao J., Liu S. (2019). Nickel hollow fiber membranes for hydrogen separation from reformate gases and water gas shift reactions operated at high temperatures. J. Membr. Sci..

[109] Cheng H., Meng B., Li C., Wang X., Meng X., Sunarso J., Tan X., Liu S. (2020). Single–step synthesized dual–layer hollow fiber membrane reactor for on–site hydrogen production through ammonia decomposition. Int. J. Hydrogen Energy.

[110] Dittrich C.J. (2020). The role of heat transfer on the feasibility of a packed–bed membrane reactor for propane dehydrogenation. Chem. Eng. J..

[111] Kyriakou V., Garagounis I., Vourros A., Vasileiou E., Manerbino A., Coors W.G., Stoukides M. (2016). Methane steam reforming at low temperatures in a BaZr_0.7_Ce_0.2_Y_0.1_O_2.9_ proton conducting membrane reactor. Appl. Catal. B Environ..

[112] Xue J., Chen Y., Wei Y., Feldhoff A., Wang H., Caro J. (2016). Gas to Liquids: Natural Gas Conversion to Aromatic Fuels and Chemicals in a Hydrogen–Permeable Ceramic Hollow Fiber Membrane Reactor. ACS Catal..

[113] Morejudo S.H., Zanon R., Escolastico S., Yuste–Tirados I., Malerod–Fjeld H., Vestre P.K., Coors W.G., Martinez A., Norby T., Serra J.M. (2016). Direct conversion of methane to aromatics in a catalytic co–ionic membrane reactor. Science.

[114] Wu Z.T., Wang B., Li K. (2011). Functional LSM–ScSZ/NiO–ScSZ dual–layer hollow fibres for partial oxidation of methane. Int. J. Hydrogen Energy.

[115] Kathiraser Y., Kawi S. (2013). La_0.6_Sr_0.4_Co_0.8_Ga_0.2_O_3−delta_ (LSCG) Hollow Fiber Membrane Reactor: Partial Oxidation of Methane at Medium Temperature. Aiche J..

[116] Li W., Cao Z., Zhu X., Yang W. (2017). High-rate hydrogen separation using an MIEC oxygen permeable membrane reactor. AIChE J..

[117] Zhu N., Zhang G., Liu Z., Dong X., Jin W., Liu S., Gur T. (2013). A Comparative Study of the Performance of SrCo_0.76_Fe_0.19_Al_0.1_O_x_ and (SrCo_0.8_Fe_0.2_O_3−δ_)_0.95_(SrAl_2_O_4_)_0.05_ Mixed–Conducting Membranes. J. Am. Ceram. Soc..

[118] Zhu N., Dong X., Liu Z., Zhang G., Jin W., Xu N. (2012). Toward highly–effective and sustainable hydrogen production: Bio–ethanol oxidative steam reforming coupled with water splitting in a thin tubular membrane reactor. Chem. Commun..

[119] Shao Z.P., Xiong G.X., Tong J.H., Dong H., Yang W.S. (2001). Ba effect in doped Sr(Co_0.8_Fe_0.2_)O_3−delta_ on the phase structure and oxygen permeation properties of the dense ceramic membranes. Sep. Purif. Technol..

[120] Tong J.H., Yang W.S., Cai R., Zhu B.C., Lin L.W. (2002). Novel and ideal zirconium–based dense membrane reactors for partial oxidation of methane to syngas. Catal. Lett..

[121] Bouwmeester H.J.M. (2003). Dense ceramic membranes for methane conversion. Catal. Today.

[122] Dong X.L., Liu Z.K., Jin W.Q., Xu N.P. (2008). A self–catalytic mixed–conducting membrane reactor for effective production of hydrogen from methane. J. Power Sources.

[123] Dong X.L., Zhang C., Chang X.F., Jin W.Q., Xu N.P. (2008). A self–catalytic membrane reactor based on a supported mixed–conducting membrane. Aiche J..

[124] Wang H., Tablet C., Feldhoff A., Caro J. (2005). A Cobalt-Free Oxygen-Permeable Membrane Based on the Perovskite-Type Oxide Ba_0.5_Sr_0.5_Zn_0.2_Fe_0.8_O_3–δ_. Adv. Mater..

[125] Li Q., Zhu X., He Y., Yang W. (2010). Partial oxidation of methane in BaCe_0.1_Co_0.4_Fe_0.5_O_3−δ_ membrane reactor. Catal. Today.

[126] Gong Z., Hong L. (2011). Integration of air separation and partial oxidation of methane in the La_0.4_Ba_0.6_Fe_0.8_Zn_0.2_O_3−δ_ membrane reactor. J. Membr. Sci..

[127] Song S., Zhang P., Han M., Singhal S.C. (2012). Oxygen permeation and partial oxidation of methane reaction in Ba_0.9_Co_0.7_Fe_0.2_Nb_0.1_O_3−δ_ oxygen permeation membrane. J. Membr. Sci..

[128] Jiang W., Zhang G., Liu Z., Zhang K., Jin W. (2013). A Novel Porous–Dense Dual–Layer Composite Membrane Reactor with Long–Term Stability. Aiche J..

[129] Liao Q., Chen Y., Wei Y., Zhou L., Wang H. (2014). Performance of U–shaped BaCo_0.7_Fe_0.2_Ta_0.1_O_3−δ_ hollow–fiber membranes reactor with high oxygen permeation for methane conversion. Chem. Eng. J..

[130] Song S., Zhang P., Zhang X., Han M. (2015). Partial oxidation of methane reaction in Ba_0.9_Co_0.7_Fe_0.2_Nb_0.1_O_3−δ_ oxygen permeation membrane with three–layer structure. Int. J. Hydrogen Energy.

[131] Meng X., Bi X., Meng B., Yang N., Tan X., Liu L., Liu S. (2017). H_2_/CH_4_/CO_2_–tolerant properties of SrCo_0.8_Fe_0.1_Ga_0.1_O_3−δ_ hollow fiber membrane reactors for methane partial oxidation to syngas. Fuel Process. Technol..

[132] Wang Z., Ashok J., Pu Z., Kawi S. (2017). Low temperature partial oxidation of methane via BaBi_0.05_Co_0.8_Nb_0.15_O_3−δ_−Ni phyllosilicate catalytic hollow fiber membrane reactor. Chem. Eng. J..

[133] Akin F., Lin Y. (2002). Oxidative coupling of methane in dense ceramic membrane reactor with high yields. AIChE J..

[134] Othman N.H., Wu Z., Li K. (2015). An oxygen permeable membrane microreactor with an in–situ deposited Bi_1.5_Y_0.3_Sm_0.2_O_3−delta_ catalyst for oxidative coupling of methane. J. Membr. Sci..

[135] Cavani F., Ballarini N., Cericola A. (2007). Oxidative dehydrogenation of ethane and propane: How far from commercial implementation?. Catal. Today.

[136] Czuprat O., Arnold M., Schirrmeister S., Schiestel T., Caro J. (2010). Influence of CO_2_ on the oxygen permeation performance of perovskite–type BaCo_x_Fe_y_Zr_z_O_3−delta_ hollow fiber membranes. J. Membr. Sci..

[137] Wang Z., Bian Z., Dewangan N., Xu J., Kawi S. (2019). High–performance catalytic perovskite hollow fiber membrane reactor for oxidative propane dehydrogenation. J. Membr. Sci..

[138] Jin Y., Meng X.X., Meng B., Yang N.T., Sunarso J., Liu S.M. (2019). Parametric modeling study of oxidative dehydrogenation of propane in La_0.6_Sr_0.4_Co_0.2_Fe_0.8_O_3−delta_ hollow fiber membrane reactor. Catal. Today.

[139] Wang H., Cong Y., Yang W. (2005). Oxidative coupling of methane in Ba_0.5_Sr_0.5_Co_0.8_Fe_0.2_O_3−δ_ tubular membrane reactors. Catal. Today.

[140] Bhatia S., Thien C.Y., Mohamed A.R. (2009). Oxidative coupling of methane (OCM) in a catalytic membrane reactor and comparison of its performance with other catalytic reactors. Chem. Eng. J..

[141] Igenegbai V.O., Almallahi R., Meyer R.J., Linic S. (2019). Oxidative Coupling of Methane over Hybrid Membrane/Catalyst Active Centers: Chemical Requirements for Prolonged Lifetime. ACS Energy Lett..

[142] Wang H., Cong Y., Yang W. (2002). Continuous Oxygen Ion Transfer Medium as a Catalyst for High Selective Oxidative Dehydrogenation of Ethane. Catal. Lett..

[143] Wang H., Tablet C., Schiestel T., Caro J. (2006). Hollow fiber membrane reactors for the oxidative activation of ethane. Catal. Today.

[144] Liang F.Y., He G.H., Jia L.J., Jiang H.Q. (2019). Cobalt–free dual–phase oxygen transporting membrane reactor for the oxidative dehydrogenation of ethane. Sep. Purif. Technol..

[145] Balachandran U., Lee T., Wang S., Dorris S. (2004). Use of mixed conducting membranes to produce hydrogen by water dissociation. Int. J. Hydrogen Energy.

[146] Park C., Lee T., Dorris S., Balachandran U. (2010). Hydrogen production from fossil and renewable sources using an oxygen transport membrane. Int. J. Hydrogen Energy.

[147] Park C., Lee T., Dorris S., Balachandran U. (2013). A cobalt–free oxygen transport membrane, BaFe_0.9_Zr_0.1_O_3−δ_, and its application for producing hydrogen. Int. J. Hydrogen Energy.

[148] Li L., Borry R.W., Iglesia E. (2002). Design and optimization of catalysts and membrane reactors for the non–oxidative conversion of methane. Chem. Eng. Sci..

[149] Liu S.M., Tan X.Y., Li K., Hughes R. (2001). Methane coupling using catalytic membrane reactors. Catal. Rev. Sci. Eng..

[150] Zhang K., Zhang G., Liu Z., Zhu J., Zhu N., Jin W. (2014). Enhanced stability of membrane reactor for thermal decomposition of CO_2_ via porous–dense–porous triple–layer composite membrane. J. Membr. Sci..

[151] Jin W., Zhang C., Chang X., Fan Y., Xing W., Xu N. (2008). Efficient catalytic decomposition of CO_2_ to CO and O_2_ over Pd/mixed–conducting oxide catalyst in an oxygen–permeable membrane reactor. Environ. Sci. Technol..

[152] Zhang C., Jin W.Q., Yang C., Xu N.P. (2009). Decomposition of CO_2_ coupled with POM in a thin tubular oxygen–permeable membrane reactor. Catal. Today.

[153] Jiang H., Xing L., Czuprat O., Wang H., Schirrmeister S., Schiestel T., Caro J. (2009). Highly effective NO decomposition by in situ removal of inhibitor oxygen using an oxygen transporting membrane. Chem. Commun..

[154] Jiang H., Wang H., Liang F., Werth S., Schiestel T., Caro J. (2009). Direct decomposition of nitrous oxide to nitrogen by in situ oxygen removal with a perovskite membrane. Angew. Chem. Int. Ed..

[155] Jiang H., Wang H., Werth S., Schiestel T., Caro J. (2008). Simultaneous production of hydrogen and synthesis gas by combining water splitting with partial oxidation of methane in a hollow–fiber membrane reactor. Angew. Chem. Int. Ed..

[156] Cao Z., Jiang H., Luo H., Baumann S., Meulenberg W.A., Voss H., Caro J. (2012). Simultaneous overcome of the equilibrium limitations in BSCF oxygen–permeable membrane reactors: Water splitting and methane coupling. Catal. Today.

[157] Alvarez Feijoo M.A., Arce Farina M.E., Suarez–Garcia A., Gonzalez–Pena D., Diez–Mediavilla M. (2019). Compounds with Epoxy Resins and Phase Change Materials for Storage in Solar Applications. Materials.

[158] Zhao L., Qi Y., Song L., Ning S., Ouyang S., Xu H., Ye J. (2019). Solar–Driven Water–Gas Shift Reaction over CuO_x_/Al_2_O_3_ with 1.1% of Light–to–Energy Storage. Angew. Chem. Int. Ed. Engl..

[159] Wang F.Q., Tan J.Y., Jin H.J., Yu L. (2015). Thermochemical performance analysis of solar driven CO_2_ methane reforming. Energy.

[160] Chuayboon S., Abanades S. (2020). An overview of solar decarbonization processes, reacting oxide materials, and thermochemical reactors for hydrogen and syngas production. Int. J. Hydrogen Energy.

[161] Li W., Zhu X., Cao Z., Wang W., Yang W. (2015). Mixed ionic–electronic conducting (MIEC) membranes for hydrogen production from water splitting. Int. J. Hydrogen Energy.

[162] Wu X.-Y., Ghoniem A.F. (2019). Mixed ionic–electronic conducting (MIEC) membranes for thermochemical reduction of CO_2_: A review. Prog. Energy Combust. Sci..

[163] Wang H., Wang B., Qi X., Wang J., Yang R., Li D., Hu X. (2021). Innovative non–oxidative methane dehydroaromatization via solar membrane reactor. Energy.

[164] Tou M., Michalsky R., Steinfeld A. (2017). Solar–Driven Thermochemical Splitting of CO_2_ and In Situ Separation of CO and O_2_ across a Ceria Redox Membrane Reactor. Joule.

[165] He R.J., Wang Y.P., Wang H.S., Lundin S.T.B., Wang B.Z., Kong H., Lu X.F., Wang J., Li W.J. (2021). A mid/low–temperature solar–driven integrated membrane reactor for the dehydrogenation of propane–A thermodynamic assessment. Appl. Therm. Eng..

[166] Kothari M., Jeon Y., Miller D.N., Pascui A.E., Kilmartin J., Wails D., Ramos S., Chadwick A., Irvine J.T.S. (2021). Platinum incorporation into titanate perovskites to deliver emergent active and stable platinum nanoparticles. Nat. Chem..

[167] Madsen B.D., Kobsiriphat W., Wang Y., Marks L.D., Barnett S.A. (2007). Nucleation of nanometer–scale electrocatalyst particles in solid oxide fuel cell anodes. J. Power Sources.

[168] Neagu D., Tsekouras G., Miller D.N., Menard H., Irvine J.T. (2013). In situ growth of nanoparticles through control of non–stoichiometry. Nat. Chem..

[169] Kousi K., Neagu D., Metcalfe I.S. (2020). Combining Exsolution and Infiltration for Redox, Low Temperature CH_4_ Conversion to Syngas. Catalysts.

[170] Neagu D., Irvine J.T.S. (2010). Structure and Properties of La_0.4_Sr_0.4_TiO_3_ Ceramics for Use as Anode Materials in Solid Oxide Fuel Cells. Chem. Mater..

[171] Myung J.H., Neagu D., Miller D.N., Irvine J.T. (2016). Switching on electrocatalytic activity in solid oxide cells. Nature.

[172] Jiang K., Liu Z., Zhang G., Jin W. (2020). A novel catalytic membrane reactor with homologous exsolution–based perovskite catalyst. J. Membr. Sci..

[173] Zhu J., Zhang G., Liu G., Liu Z., Jin W., Xu N. (2017). Perovskite Hollow Fibers with Precisely Controlled Cation Stoichiometry via One–Step Thermal Processing. Adv. Mater..

